# Artificial Intelligence to Decode Cancer Mechanism: Beyond Patient Stratification for Precision Oncology

**DOI:** 10.3389/fphar.2020.01177

**Published:** 2020-08-12

**Authors:** Sandip Kumar Patel, Bhawana George, Vineeta Rai

**Affiliations:** ^1^Department of Biosciences and Bioengineering, Indian Institute of Technology Bombay, Mumbai, India; ^2^Buck Institute for Research on Aging, Novato, CA, United States; ^3^Department of Hematopathology, The University of Texas MD Anderson Cancer Center, Houston, TX, United States; ^4^Department of Entomology & Plant Pathology, North Carolina State University, Raleigh, NC, United States

**Keywords:** multi-omics, artificial intelligence (AI), data integration, cancer biomarkers, patient stratification

## Abstract

The multitude of multi-omics data generated cost-effectively using advanced high-throughput technologies has imposed challenging domain for research in Artificial Intelligence (AI). Data curation poses a significant challenge as different parameters, instruments, and sample preparations approaches are employed for generating these big data sets. AI could reduce the fuzziness and randomness in data handling and build a platform for the data ecosystem, and thus serve as the primary choice for data mining and big data analysis to make informed decisions. However, AI implication remains intricate for researchers/clinicians lacking specific training in computational tools and informatics. Cancer is a major cause of death worldwide, accounting for an estimated 9.6 million deaths in 2018. Certain cancers, such as pancreatic and gastric cancers, are detected only after they have reached their advanced stages with frequent relapses. Cancer is one of the most complex diseases affecting a range of organs with diverse disease progression mechanisms and the effectors ranging from gene-epigenetics to a wide array of metabolites. Hence a comprehensive study, including genomics, epi-genomics, transcriptomics, proteomics, and metabolomics, along with the medical/mass-spectrometry imaging, patient clinical history, treatments provided, genetics, and disease endemicity, is essential. Cancer Moonshot℠ Research Initiatives by NIH National Cancer Institute aims to collect as much information as possible from different regions of the world and make a cancer data repository. AI could play an immense role in (a) analysis of complex and heterogeneous data sets (multi-omics and/or inter-omics), (b) data integration to provide a holistic disease molecular mechanism, (c) identification of diagnostic and prognostic markers, and (d) monitor patient’s response to drugs/treatments and recovery. AI enables precision disease management well beyond the prevalent disease stratification patterns, such as differential expression and supervised classification. This review highlights critical advances and challenges in omics data analysis, dealing with data variability from lab-to-lab, and data integration. We also describe methods used in data mining and AI methods to obtain robust results for precision medicine from “big” data. In the future, AI could be expanded to achieve ground-breaking progress in disease management.

## Introduction

Artificial intelligence (AI) is a branch of computer science with enhanced analytical or predictive capabilities to perform interdisciplinary tasks that otherwise require human intellect. AI has intensive problem-solving capabilities including prediction, data scalability, dimensionality, and integration, reasoning about their underlying phenomena and/or big data transformation into clinically actionable knowledge, based on the learning from model data sets. The learning capacity is maximized by improving the prediction task based on problem-speciﬁc measurements of performance. Particularly, machine learning (ML) and deep learning (DL)-based approaches were gaining recognition and emerged as key components in biomedical data analysis, driven by health care data availability and rapid progress of analytics techniques (Jiang et al., 2017; Saltz et al., 2018; Huang et al., 2020; Ibrahim et al., 2020). AI is currently used to automate the information extraction, summarize the electronic medical records or hand-written doctor notes, integrate health records, and store information in cloud scaling (big data storage) (Bedi et al., 2015; Chang et al., 2016; Miotto et al., 2016; Osborne et al., 2016; Garvin et al., 2018; Syrjala, 2018) AI has immense potentials to contribute significantly at every stage of cancer management ranging from reliable early detection, stratification, determination of infiltrative tumor margins during surgical treatment, response to drugs/therapy, tracking tumor evolution and potential acquired resistance to treatments over time, prediction of tumor aggressiveness, metastasis pattern, and recurrence (Bi et al., 2019).

**Figure 1:**
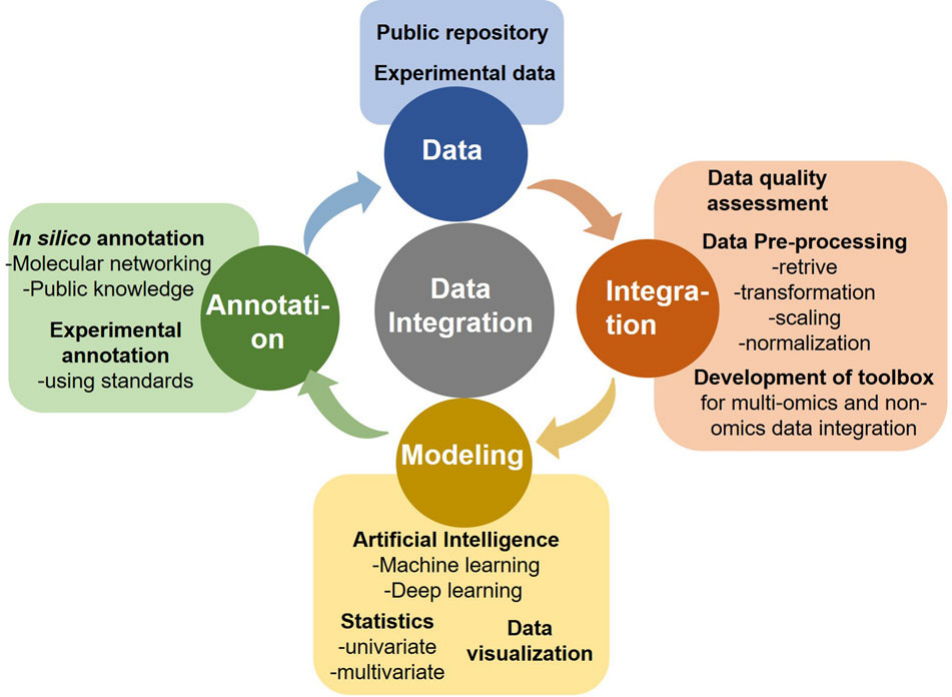
Components of omics data analytics.

Cancer is a major cause of death worldwide, accounting for an estimated 9.6 million deaths in 2018. Cancers can originate from various organs viz. lung, breast, kidney, represent phenotypic diversity like cell surface markers, molecular mutations (p53, PTEN, ER), demonstrate varied growth rate and apoptosis based on the cancer microenvironment and status of blood supply, and its aggressive nature. Also, cancer has a diverse disease progression mechanism and the effectors ranging from gene-epigenetics to a wide array of metabolites. Cancer/tumor being highly heterogeneous in terms of inter-tumor heterogeneity (cancers from different patients) and intra-tumor heterogeneity (within a single tumor) impose challenges for both detection, treatments, and recurrence. Medical decisions for cancer treatment should consider not only its variegated forms with the evolution of disease but also the individual patient’s condition and their ability to receive and respond to treatment. Certain cancers, such as pancreatic and gastric cancers, are detected only after they have reached their advanced stages with frequent relapses. Integration of “multi-omics” (genomics, epi-genomics, transcriptomics, proteomics, and metabolomics), and “non-omics” (medical/mass-spectrometry imaging, patient clinical history, treatments, and disease endemicity) data could help overcome the challenges in the accurate detection, characterization, and monitoring of cancers. AI could play an immense role in the analysis of complex and heterogeneous data sets, particularly from multi-omics and inter-omics approaches and data integration to provide a holistic disease molecular mechanism, identification of novel dynamic diagnostic and prognostic markers and enable precision cancer management, well beyond the prevalent disease stratification patterns such as differential expression, and supervised classification (**Figure 1**). Advanced computational analyses could also augment a global interpretation and automation of the cancer patient radiographs that most commonly relies upon visual evaluations and hence differ in disease assessments. Cancer Moonsho℠ Research Initiatives by NIH National Cancer Institute aims to collect as many omics and non-omics information as possible from different regions of the world to create a national ecosystem for sharing and analyzing cancer data (Cancer Moonshot - National Cancer Institute, 2016). The project will help develop human tumor atlas, predict response to standard treatments, optimize guidelines for systematic cancer prediction and treatments, and identify ways to overcome drug resistance to improve (i) current understanding of cancer, (ii) enable new strategies/technologies for cancer characterization, (iii) early detection of tumors/cancer, and (iv) extend therapies to more patients in a personalized manner (Cancer Moonshot - National Cancer Institute, 2016). The large multidimensional biological data sets (including individual variability in genes, function, and environment) generated, and/or compiled for the fulfillment of this cross-border project require advanced computational analysis, and AI certainly could be one of the key plays.

Recently AI is successfully applied to tumor image segmentation, identify, and quantify the rate and amount of mitosis (Romo-Bucheli et al., 2017), screening mutations (Coudray et al., 2018), auto-detect and classify benign nuclei from cancer cells (Sirinukunwattana et al., 2016; Xu et al., 2016), protein alignments and spatial localization (Saltz et al., 2018), predicting unknown metabolites, precision medicine matching trials (Korbar et al., 2017; Coudray et al., 2018), drug repurposing (Aliper et al., 2016), liquid biopsies and pharmacogenomics based cancer screening/monitoring and predicting the patient outcomes (Cohen et al., 2018; Low et al., 2018), drug discovery (Abadi et al., 2017; Yu P. et al., 2017) and so on. AI has outperformed pathologists and dermatologists in diagnosing metastatic breast cancer (Low et al., 2018) and melanoma (Bejnordi et al., 2017). Conversely, multi-omics data has immense potentials to identify the caveat in the current AI-based cancer diagnostics, stratification, mutant identification, treatment, and drug repurposing approaches, which could advance precision oncology research (Li et al., 2018). However, we have limited knowledge in the multi-omics and inter-omics data analysis and availability of algorithms (Buchhalter et al., 2014). This review highlights the current AI application in data integration, advancement, scope, and challenges in oncology research and clinical use. The reports mostly cover the articles published in the last two decades (2000–2020).

## Implications of Artificial Intelligence in Cancer Multi-Omics

Advancements in multidimensional “omics” technologies ranging from next-generation sequencing to the mass spectrometry have led to a plethora of information. AI mediated data integration obtained from different “-omics” platforms such as genomics, epigenomics, transcriptomics, proteomics, and metabolomics enables the understanding of complex biological systems by describing nearly all biomolecules ranging from DNA to metabolites. Multi-omics researches have diverse applications in veterinary medicine (Li Q. et al., 2015), microbiology (Zhang et al., 2010), agriculture science (Van Emon, 2016), biofuel (Rai et al., 2016), and biomedical sciences (More et al., 2015; Hasin et al., 2017; Awasthi et al., 2018; Patel et al., 2019) including oncology (see **Table 1**).

**Table 1:** Comprehensive list of Artificial Intelligence-based omics and non-omics investigations in oncology.

#	Type of omics	Data type	AI	Tools/analysis	Type of cancer	References
1	Non-omics	Clinicopathological	**DL**	Genetic algorithm and Pearson’s correlation coefficient	Oral	(Chang et al., 2011)
2		Clinicopathological	**DL**	Neural network	Colorectal	(Bottaci et al., 1997)
3		Clinicopathological	**DL**	Decision tree, artificial neural network (ANN), support vector machine (SVM) and logistic regression	Colorectal	(Wang et al., 2019)
4		Clinicopathological	**DL**	ANN and Cox regression	Gastric	(Zhu et al., 2013)
5		Clinicopathological	**DL**	Cox proportional hazard and ANN	Gastric	(Biglarian et al., 2011)
6		Sonographic images	**DL**	Deep convolutional neural network (DCNN)	Thyroid	(Li X. et al., 2019)
7		MR images	**DL**	Faster region-based convolutional neural networks (Faster R-CNN)	Metastatic lymph nodes	(Lu Y. et al., 2018)
8		Dermoscopic images	**DL**	Convolutional neural networks (CNN)	Melanoma	(Haenssle et al., 2018)
9		Digital Mammography DREAM	**DL**	Faster region-based convolutional neural networks (Faster R-CNN)	Breast	(Ribli et al., 2018)
10		Clinicopathological	**ML**	Neural networks, decision trees, and logistic regression	Breast	(Delen et al., 2005)
11		Clinicopathological	**ML**	ANN, SVM, and semi-supervised learning	Breast	(Park et al., 2013)
12		Clinicopathological	**ML**	Extreme Learning Machine (ELM), Neural networks and Genetic algorithm	Prostate	(Jović et al., 2017)
13		Clinicopathological	**ML**	Two-stage fuzzy neural network	Prostate	(Kuo et al., 2015)
14		Clinicopathological	**ML**	Linear regression, Decision Trees, Gradient Boosting Machines, and Support Vector Machines	Lung	(Lynch et al., 2017)
15		Radiomics	**ML**	Decision tree, AdaBoost, algorithm, RUSBoost algorithm, matthews correlation coefficient (MCC)	Gliomas	(Lu C. F. et al., 2018)
16		MR images & Clinicopathological	**ML**	SVM, bagged SVM, K-nearest neighbor (KNN), adaptive boosted trees (AdaBoost), random forest (RF), and gradient boosted trees (GBT)	Bladder	(Hasnain et al., 2019)
17	Single omics	Genomics	**DL**	Prognosis-enhanced neural networks (ENN), SVM, and probabilistic-enhanced NN (PENN)	Pan Cancer	(Vasudevan and Murugesan, 2018)
18		Proteomics	**DL**	SVM and C4.5	Breast	(Karsan et al., 2005)
19		Proteomics	**DL**	Deep Learning neural network (DLNN)	Myeloid Leukemia	(Liang et al., 2019)
20		Metabolomics	**DL**	multiple logistic regression (MLR) and alternative decision tree (ADTree)	Breast	(Murata et al., 2019)
21		Genomics	**ML**	SVM, genetic algorithm, log-rank test, and Cox hazard regression model	Ovarian	(Lu et al., 2019)
22		Genomics	**ML**	Restricted Boltzmann Machine (RBM), Deep Belief Network (DBN), and Pathway based Deep Clustering model (PACL)	GBM and ovarian cancer	(Mallavarapu et al., 2019)
23		Metabolomics	**ML**	SVM, Naive Bayes, Partial Least Square Discriminant Analysis (PLS-DA), LASSO, RF, KNN, and C4.5	Colonic	(Eisner et al., 2013)
24		Metabolomics	**ML**	RF, SVM, recursive partitioning and regression trees (RPART), linear discriminant analysis (LDA), prediction analysis for microarrays (PAM), and generalized boosted models (GBM)	Breast	(Alakwaa et al., 2018)
25	Non-omics and single omics	MR images and genomics	**DL**	Residual convolutional neural network (RCNN)	Gliomas	(Chang et al., 2018)
26		Clinicopathological and genomics	**DL**	DNN, Multi modal Deep Neural Network by integrating Multiulti-dimensional Data (MDNNMD) and receiver operating characteristic (ROC)	Breast	(Sun et al., 2018)
27		Clinicopathological and genomics	**ML**	Ensemble models-SVM, ANN, KNN, ROC, and calibration slope (CS).	Breast	(Zhao et al., 2018)
28		Clinicopathological and genomics	**ML**	SVM, and ROC	Prostate	(Zhang et al., 2017)
29		Histopathology images and proteomics	**ML**	RF and CNN	Kidney	(Azuaje et al., 2019)
30	Multi-omics	Epigenetics, genomics, and transcriptomics	**DL**	Hierarchical integration deep flexible neural forest framework (HI-DFNForest), KNN, SVM, RF, and multi-grained cascade forest (gcForest)	BRCA, GBM, and OV	(Xu et al., 2019)
31		Epigenetics and transcriptomics	**DL**	Unsupervised feed-forward, nonrecurrent neural network, Cox proportional hazards (Cox-PH) model, K-means clustering, SVM algorithm, concordance index, Log-rank P-value of Cox-PH regression, Brier score, and ANOVA test F values	Liver	(Chaudhary et al., 2018)
32		Epigenetics and transcriptomics	**DL**	OmiVAE, k-means clustering, support vector machine, Variational autoencoder (VAE), PCA, t-SNE, KPCA, and UMAP	Pan cancer	(Zhang X. et al., 2019)
33		Epigenetics and transcriptomics	**DL**	DeepProg, Autoencoder, Cox-PH model, Gaussian mixture model, concordance index, and Wilcoxon rank-sum test	Pan cancer	(Poirion et al., 2019)
34		Genomics, transcriptomics, and proteomics	**ML**	Generic model, gene-specific model, RF, Random Forest Regressor, and trans-tissue model, Wilcoxon signed-rank test	Breast and ovarian	(Li H. et al., 2019)

### Genomics

Genomics data analysis relies on the nucleotide sequences, including expressed sequence tags (ESTs), cDNAs, and gene arrangements on the respective chromosomes. Rapid advances in the next‐generation sequencer (NGS) (Paolillo et al., 2016) and *in silico* computational algorithms have led to high-throughput data generation for whole genomes sequencing (WGS) and epigenomes. WGS comprehensively explores all types of genomic alterations in cancer and provides information on the repertoire of driver mutations and mutational signatures (including non‐coding regions) in cancer genomes, which remain widely unexplored. Ley et al. reported the first-ever WGS analysis of cancer (cytogenetically normal acute myeloid leukemia, AML) (Ley et al., 2008), merely 6 months post-publication of the first human whole-genome sequence (Wheeler et al., 2008). Since then several cancer genomics databases and projects including The Cancer Genome Atlas (TCGA) (Wang Z. et al., 2016), the International Cancer Genome Consortium (ICGC) (Zhang J. et al., 2019), Catalog of Somatic Mutations in Cancer (COSMIC) (Forbes et al., 2015), Cancer Genomic Hub (CGHub) (Wilks et al., 2014), Therapeutically Applicable Research to Generate Effective Treatments (TARGET) (Therapeutically Applicable Research to Generate Effective Treatments (TARGET)), cBioPortal (Gao et al., 2013), MethyCancer (He et al., 2008), UCSC Cancer Genomics Browser (Goldman et al., 2013) and moonshot project (Cancer Moonshot - National Cancer Institute, 2016) have surfaced (also see **Table 2**). Data accessibility has further led to the development of tools and resources to facilitate the rapid detection and analysis of biologically relevant genomic outcomes (Cerami et al., 2012; Gao et al., 2013; Gonzalez-Perez et al., 2013; Rubio-Perez et al., 2015; Chakraborty et al., 2018). WGS is thus a powerful tool to understand cancer genomics that typically contains unpredictable numbers of point mutations, fusions, and other aberrations. In contrast, targeted approaches like whole-exome sequencing (WES) are easier to analyze but miss out information of untranslated, intronic, and intergenic regions, which might have an impact on the molecular pathogenesis of cancer (Nik-Zainal et al., 2016). However, there are several associated limitations: (i) a vast majority of cancer genomics efforts remain focused around targeted approaches viz. WES (Morris et al., 2017) (ii) many of the genomics data reported lacks a comprehensive clinical annotation required for linking genomic events to specific cancer types, prognoses, and treatment responses (Robinson et al., 2017) (iii) most of the preliminary studies are performed on untreated cancers, and thus do not provide insight into the response to treatment regimens (Robinson et al., 2017). Integrating the cancer genomics data with clinical physiology data could, therefore, be expected to better define cancer biology and responses to treatments. Several studies have integrated genomics and non-omics cancer data (see **Table 1**). Histopathological images integration with genomics helps retrieves better information on cancer tissue architecture, which is generally compromised in molecular assays, rendering this rich information underused (López de Maturana et al., 2019). AI algorithms classify breast cancers using prognostic factors to quantitative image (Yuan et al., 2012) and the public data set (TCGA) (Yuan et al., 2012). AI algorithms to integrate (multi-) omics data with the pathology images has been successfully extended to develop predictive models for prostate cancer (Robinson et al., 2015), renal cell carcinoma (Schoof et al., 2019), low-grade glioma (Brat et al., 2015), and non-small cell lung cancer (Yu et al., 2016). Alongside integrating the multi-omics data from different platforms, transcriptomics, proteomics, and metabolomics with genomics could consolidate molecular information. Wu P. et al., 2019 integrated the Clinical Proteomic Tumor Analysis Consortium (CPTAC) mass spectrometry-based proteomics data for selected breast, colon, and ovarian tumors with TCGA into the cBioPortal (cBioPortal for Cancer Genomics) to support easy exploration and integrative analysis of the proteomic data sets in the context of the clinical and genomics data from the same tumors (Wu P. et al., 2019). Considering the diversity of cancer genomes and phenotypes, cataloging and interpretation of the abundant mutation, particularly non‐coding and structure variants, could be performed with confidence *via* integrating clinicopathological information along with transcriptomics, and epigenomics to decide the precise treatments that will produce the best results for the cancer patients.

**Table 2:** List of cancer genomics databases.

**#**	Cancer genomic database name	Cancer alteration types	Organisms	Public data
1	The Cancer Genome Atlas (TCGA)	Copy number, mutation, methylation, gene expression, miRNA expression	Human	Yes
2	The International Cancer Genome Consortium (ICGC)	Mutation	Human	Yes
3	Catalog of Somatic Mutations in Cancer (COSMIC)	Mutation	Human	No
4	cBio Cancer Genomics Portal	Copy number, mutation, methylation, gene expression, miRNA expression, protein, phosphorylation	Human	Yes
5	MethyCancer	Methylation	Human	Yes
6	MutaGene	Mutation	Human	Yes
7	Moonshot project	Copy number, gene expression	Human	Yes
8	Integrative Oncogenomics Cancer Browser (IntOGen)	Copy number, mutation, gene expression	Human	Yes
9	Mouse Retrovirus Tagged Cancer Gene Database	Mutation	Mouse	Yes
10	Mouse Tumor Biology Database	Copy number, mutation, methylation, gene expression	Mouse	No
11	OncoDB.HCC	Copy number, gene expression, QTL	Human, mouse, rat	No
12	UCSC Cancer Genomics Browser	Copy number, mutation, gene expression, miRNA	Human, mouse, rat	Yes

### Transcriptomics

Transcriptome denotes the active genes as well as long-noncoding RNA, short RNAs such as microRNAs, small nuclear RNAs in a defined physiological condition. The system-wide transcriptomic analysis evaluates overall transcripts in a metabolic process, while the targeted approach provides information regarding known genes. Differential expression of protein-coding RNA could provide insight into the disease mechanism, as well as integrated with genomics and proteomics to discover novel genes and their functional relevance. While non-coding RNAs have regulatory functions in several metabolic diseases, neurological disorders, and cancer. Transcriptome is directly co-related to any epigenomic change that manifests cancer, hence the integration of epigenomics and transcriptomics data could extend our understanding of cancer biology such studies are reported in breast (Robinson et al., 2015), prostate cancer (Varambally et al., 2002; Bhasin et al., 2015), head and neck squamous cell carcinoma (HNSCC) (Kelley et al., 2017). Also, the transcriptomics and epigenomics data integration approach opens-up avenues to know more about the promoter crosstalk through a shared enhancer (Eun et al., 2013) and dynamic switching of promoter and enhancer domains (Sohni et al., 2015). Moarii et al. used a large data set of 672 cancerous and healthy methylomes gene expression and copy number profiles from TCGA and performed a meta-analysis to clarify the interplay between promoter methylation and gene expression in normal and cancer samples (Moarii et al., 2015). Vantaku et al. demonstrated a novel approach for the unbiased integration of transcriptomics, metabolomics, lipidomics, and data to robustly predict high-grade patient survival and discovery of novel therapeutic targets in bladder cancer (Vantaku et al., 2019).

### Proteomics

Proteomic profiles reveal cellular/molecular responses to (epi-) genomics, and environmental alterations, and their feedback responses. Post-translation modifications, including phosphorylation, glycosylation, ubiquitination, nitrosylation, enrich the protein repertoire (protein isoforms), and impacts protein functions like transport, enzymatic activity, and intracellular signaling pathways in cancer. Classifying specific protein isoforms provide unmatched clinical sensitivity and specificity. Various tissue and plasma proteomics studies are performed (Peng et al., 2018) to screen and diagnose cancers including colorectal (Tsai et al., 2012; Fayazfar et al., 2019; Thorsen et al., 2019), breast(Mishra et al., 2015), liver (Yang et al., 2013), oral (Lai et al., 2010) and so on. MS has applications beyond disease diagnostics and could be extended to monitor the feedback responses towards therapy, identify drug toxicity, and discovering new biomarkers. High-quality data sets are obligatory for clinical MS. Hence improvements in MS-instrument quality and robustness, automated sample processing, robust data analysis pipelines, and online automation (cloud computing) to synchronize results, data sets, and data portability have contributed to expanding the use and impact of MS in cancer research. Also, to deal with the variations in the proteomics data sets across the globe, Proteomics Standards Initiative (PSI) from the Human Proteome Organization (HUPO) has setup guidelines for sample collection viz. selecting appropriate disease controls, categorizing disease and sub-disease status (Maes et al., 2015), storage to rule-out pre-analytical variables (including patient and instrumental factors) that contribute to a large extent of variation, calibrating MS instrument for data-quality assurance, data reporting for untargeted (Martínez-Bartolomé et al., 2014) and targeted (Abbatiello et al., 2017) analysis. An amalgamation of proteomics data with (epi-)genomics, transcriptomics, metabolomics, and cancer histopathological images using AI gives confidence in the data or metabolic pathways identification. Proteomics investigation of breast cancer contoured more than 12,000 proteins and 33,000 phospho-sites. Proteogenomic analysis associated DNA mutations (data obtained from TCGA) to protein signaling to pinpoint the genetic drives of cancer, and revealed new signaling pathways for the breast cancer subtypes with specific mutations (PIK3CA and TP53) and identified two candidate markers (SKP1 and CETN3) in basal-like breast cancer (Mertins et al., 2016). Liu et al., integrated transcriptome (RNA-seq) and proteome (data-independent acquisition, DIA) data to co-relating RNA splicing links isoform expression with proteome diversity that may help for studying the perturbations associated with cancer (Liu et al., 2017). MS imaging (MSI) is yet another advancement in MS that enables visualization of tumor microenvironmental biochemistry and empowers tumor biology investigation to an entirely novel biochemical perspective, thereby potentially leading to the identification of a new pool of cancer biomarkers (Bi et al., 2019). High-throughput MSI analysis is a powerful tool for biomarker identification in a spatial manner, tracking drugs and its metabolites, imaging drug-response at cellular-level. MSI tool was used to identify unique region-of-interest–specific biomarkers (lipid signature) and therapeutic targets to classify colorectal cancer and subtyping in non-small cell lung cancer (Kriegsmann et al., 2016). MSI also finds application in the identification of prognostic signatures beyond classical histology. Proteins and protein isoforms associated with patient survival in four different high-grade sarcoma subtypes (Lou et al., 2017) and colorectal adenocarcinoma (Hinsch et al., 2017) were identified. In gastric adenocarcinoma, native glycan fragments detected by MALDI-FT-ICR mass spectrometry imaging were linked to patient prognosis (Kunzke et al., 2017). Combining MSI with histology enables the extraction of molecular proﬁles from speciﬁc regions of tissue or histopathological entities, implying MSI can facilitate intelligent knife (iKnife) in sorting tumors during surgery with high sensitivity and speciﬁcity (Balog et al., 2013). Certainly, MS-based analysis, along with histopathological diagnosis, can show a stronger association with the clinical outcome (Huber et al., 2014). Recently MSI data is combined with other imaging data like fluorescence *in situ* hybridization, tissue microarrays, confocal Raman spectroscopy, and MRI, for example, MRI and MSI imaging data were collated to analyze brain pathophysiology (Porta Siegel et al., 2018). Combing vasculature staining (using an anti-CD31 antibody) and MSI could help attain a better picture of vascularization as well as vessel characteristics. However, with emerging MS technologies, there are still challenges in its clinical application including nonoptimized raw data preprocessing, imprecise image co-registration, and limited pattern recognition capabilities due to lack of reference spectra database (Addie et al., 2015). Nevertheless, efforts/measures are taken towards the successful implementation of MS technology for diagnosis of cancer biomarkers translatable to clinical setting. Additionally, the imaging data could be integrated with LC/GC-MS, the workhorse technique of proteomics workflow that includes the extraction of total proteins/peptides, fractionation, and deep proteomic analysis. Delcourt et al., combined MSI and top-down microproteomics to detect potential protein markers in serous ovarian cancer (Delcourt et al., 2017). Using LC-MS and peptide fractionation Kulak et al. achieved deep coverage of cellular proteomes with sub-microgram sample input (Liebl, 1967). Further the cancer signature biomarkers could be used to stratify patients according to subtype, metastatic risk, progression, recurrence, and treatment response. Lately single-cell proteomics is gaining importance to bring comprehensive insights into the cancer heterogeneity, clonality to metastasis or to capture information from rare/mutated cells (Doerr, 2019). Using a quantitative single-cell proteomics approach Schoof et al., characterized an acute myeloid leukemia hierarchy (Schoof et al., 2019).

### Metabolomics

Metabolomics is a systematic analysis of small molecules (<1kD) within cells, biofluids, tissues, or organisms involved in primary or secondary metabolic processes. Metabolites (small molecules) are highly diverse classified into multiple categories: amino acids, lipids, nucleotides, carbohydrates, and organic acids. Metabolite repertoire changes significantly during the process of normal growth and development and/or exposure to stress, allergens, and disease conditions (Bertini et al., 2009; Lin et al., 2011; Veselkov et al., 2011), which relates strongly to the final clinical phenotype. Metabolomics thus enhances our molecular understanding of disease mechanisms, progression, response to drugs/treatments, and recurrence probability. Typical metabolomics analysis workflow comprises of metabolite extractions, separation by liquid/gas chromatography, capillary electrophoresis and ion mobility, detection by mass spectrometry (MS), or nuclear magnetic resonance (NMR) spectroscopy and data analysis. MS applications in metabolomics have increased exponentially since the discovery and development of soft ionization tools like electrospray ionization (ESI) and matrix-assisted laser desorption ionization (MALDI). Several separation-free MS techniques including direct infusion-MS, MALDI-MS, mass spectrometry imaging (MSI), and direct analysis in real-time mass spectrometry are gaining popularity. The advantages of separation-free mass spectrometry are reduced sample volume requirements and minimization of the analytical variation. Untargeted metabolomics approaches are ideally used for hypothesis development, as it simultaneously identifies several unknown/known metabolites and quantifies. However, diverse physical and chemical properties and wide concentration ranges of the metabolites, biological variations(Heinemann et al., 2014), and identification of the unknown compounds based on the MS/MS fragmentation patterns impose challenges for untargeted metabolomics. For a long time, researchers have identified the unknows in the biological samples by complementing the MS/MS fragmentation with public repository or standards, which leads to the identification of a very limited number of metabolites, while a majority of the potentially useful information in MS/MS data sets remains uncurated. Molecular networking like GNPS has proved to be very useful in cataloging the uncurated MS/MS data sets *via* a spectral correlation and visualization approach that can detect sets of spectra from related molecules even when the spectra themselves are not matched to any known compounds (Wang M. et al., 2016). ML in combination with data mining algorithms (supervised and unsupervised) like principal component analysis or hierarchical clustering has transformed metabolomics studies like analyzing several variables/treatments simultaneously (Duan et al., 2005; Bertini et al., 2009; Guan et al., 2009). Particularly unsupervised data mining allows extracting meaningful relationships between samples with less risk of human bias. Metabolomics is applied in biomarker identification for diagnosis, monitoring, and prognosis of several diseases(Alvarez et al., 2017; Chorell et al., 2017; Perng et al., 2017; Patel et al., 2019), particularly those impacting metabolic functions, such as cancer. Metabolomic biomarkers for several cancers including colorectal (Ma et al., 2012; Nishiumi et al., 2012; Manna et al., 2014; Yamazaki, 2015) pancreatic (Zhang et al., 2012), lungs (Koutros et al., 2013; Li et al., 2014; Zhuang et al., 2016), breast (Cui et al., 2016; Li et al., 2020), gastric (Ikeda et al., 2012), ovarian (Zhang et al., 2013), and prostate (Koutros et al., 2013; Mondul et al., 2014; Kelly et al., 2016) have been reported. Despite numerous ongoing studies, limited metabolomics biomarkers reach clinical trials, implying improvements in experimental designs, data analysis with reduced false discovery rates, pinpointing molecules accountable for metabolic aberrations, and data interpretation is needed. Besides, we also must overcome interlaboratory variability by generalizing the protocols that are robust and adaptable to enhance reproducibility. Indeed, MS-based metabolomics biomarker discoveries have entered the new realms of MSI that present intuitive metabolites distribution in tissues or cells. MSI is performed in two modes, namely, imaging(Stoeckli et al., 2001) to correlate with histology and profiling, to know the overall metabolites (Cornett et al., 2006). MSI alone or in conjunction with (immuno-)histochemistry (IHC) enhances our understanding of complex heterogeneous cancer metabolic reprogramming with spatial information and facilitate the discovery of potential metabolic vulnerabilities that might be targeted for tumor therapy. MSI suffers some technical limitations like area of detection limits, instrument sensitivity at the high spatial resolution, ion suppression, matrix effects, and data analysis, particularly normalization and background correction, but has tremendous potential to improve cancer diagnostics. Huang et al. developed a graphical data processing pipeline for MSI based spatially resolved metabolomics (Huang et al., 2019), which could achieve multivariate statistical results in an intuitive and simple way as well as discovery low-abundant but reliable biomarkers in heterogeneous tumors. MSI has been employed to different cancers including brain (Jarmusch et al., 2016; Clark et al., 2018), breast (Guenther et al., 2015; Abdelmoula et al., 2016; Angerer et al., 2016; Wang S. et al., 2016; Torata et al., 2018; Vidavsky et al., 2019), lung (Calligaris et al., 2015; Li T. et al., 2015; Carter et al., 2017; Holzlechner et al., 2018), ovarian (Dória et al., 2016; Briggs et al., 2019), prostrate (Wang et al., 2017), esophageal (Guo et al., 2014; Abbassi-Ghadi et al., 2016; Sun et al., 2019a), colon (Hiraide et al., 2016; Inglese et al., 2017), oral (Uchiyama et al., 2014; Bednarczyk et al., 2019), skin (Xu et al., 2017; Margulis et al., 2018), adrenal gland (Sun et al., 2019b) and gastrointestinal stromal tumors (Abu Sammour et al., 2019) for spatial metabolomics analysis. MSI is also used to determine the metabolite changes in the 3D osteosarcoma cell culture model upon drug treatments (Palubeckaitė et al., 2020). MSI has been used to investigate tumor biopsy tissues for hypoxia (Chughtai et al., 2013; Jiang et al., 2015), driver of tumor resistance to radiotherapy or chemotherapy, and lipid distributions (Inglese et al., 2017; Paine et al., 2019). Esteva et al., employed deep convolutional neural networks (CNNs)-representing a diverse class of multi-layer artificial neural networks, pre-trained on millions of images representing more than 1000 generic image classes to automate the classification of skin cancers (Esteva et al., 2017). The same approach could be extrapolated for analyzing the images captured by MSI for better cancer management. Inglese et al. recently developed a new computational multimodal pipeline Spatial Correlation Image Analysis (SPACiAL) to integrate MSI molecular imaging data with multiplex IHC. The pipeline allows comprehensive analyses of metabolic heterogeneity, thereby increasing the efficiency and precision for spatially resolved analyses of specific cell types (Inglese et al., 2017).

## Consideration and Challenges For Ai-Mediated Multi-Omics Data Integration

**Figure 2:**
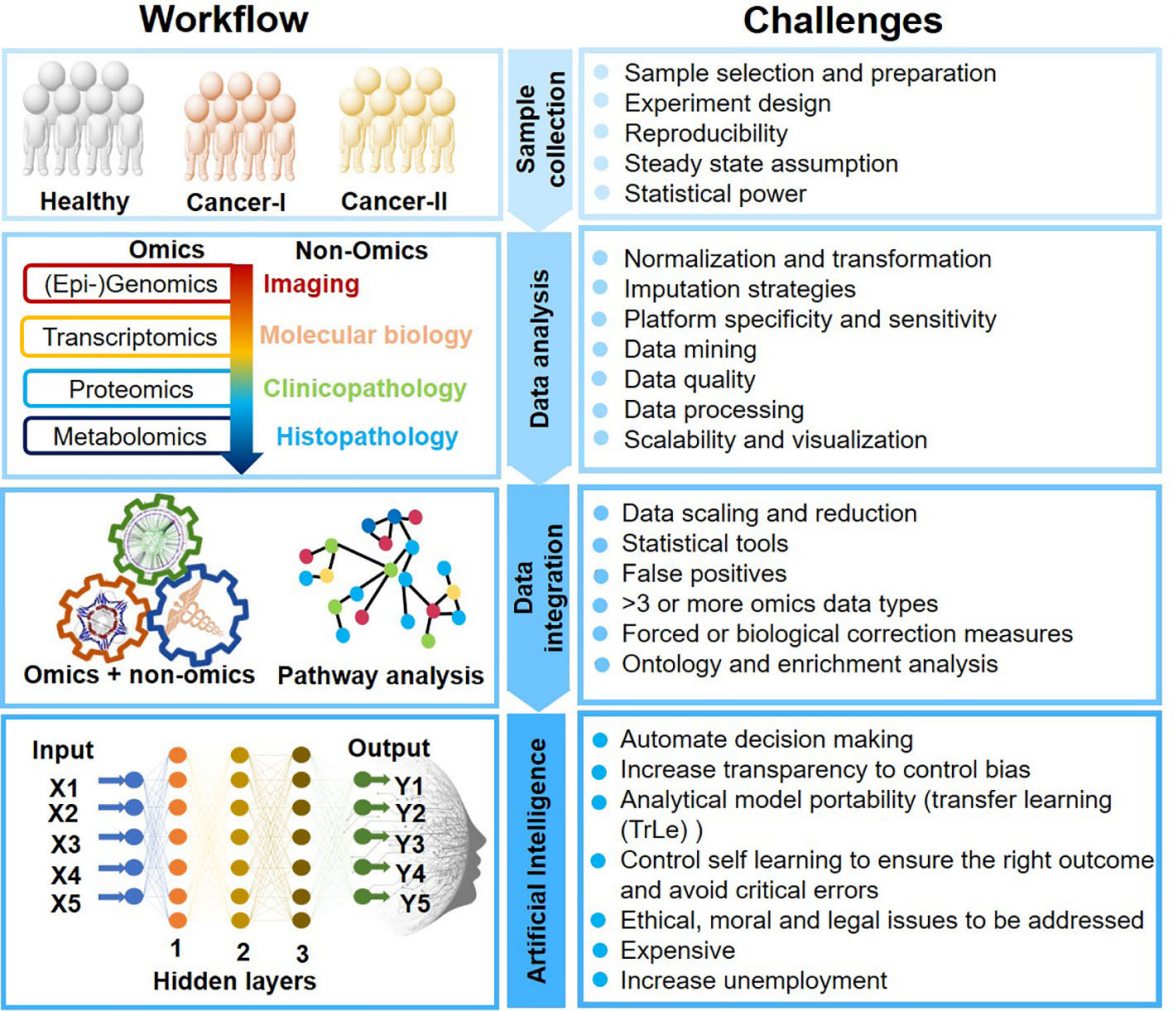
Artificial Intelligence-mediated oncology workflow and challenges.

AI-mediated clinical cancer research has attained new heights for its unpreceded learning capabilities to process complex data. ML and deep learning (DL) are the subset of artificial intelligence that enables computers to learn with data without being explicitly programmed. AI analytical skills are primarily due to image recognition, computer vision, data integration, decision making, and natural language processing. AI could thus self-adapt, synchronize qualitative and quantitative information, and validate clinical results obtained from multiple platforms. However, AI applications in oncology research still is infancy and must overcome several challenges (**Figure 2**).

### Data Integration: A Major Challenge in Precision Oncology

**Figure 3:**
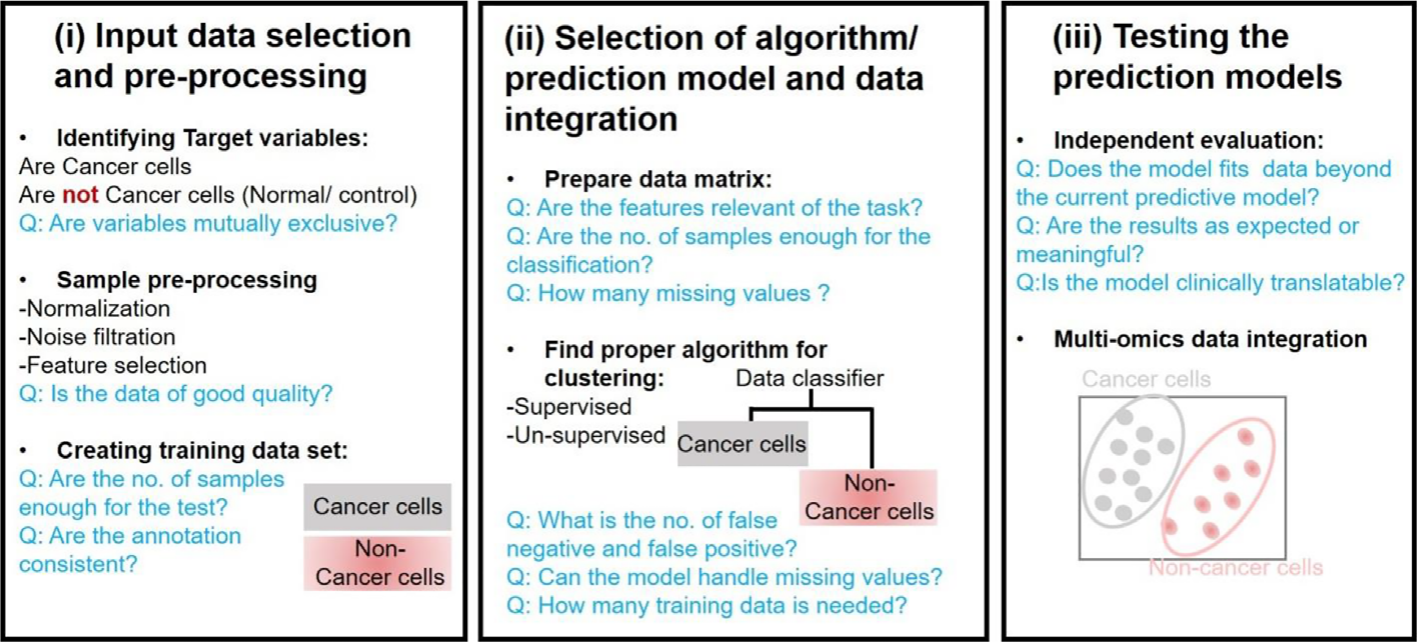
Considerations for major stage of Machine Learning based analysis in oncology.

A major challenge in precision oncology is to integrate data generated from multiple types of omics to predict biomarkers or phenotypic outcomes (tumor/normal, early/late stage, survival, etc.). Machine learning tasks consist of three key steps in order to develop a computational model for biological data integration and analysis: (i) selection and pre-processing of data set, (ii) selection of algorithm and identify the ways to train it for development of a prediction model, and (iii) validation of the model in another data set (**Figure 3**).

#### Input Data Selection and Pre-Processing

Input data for most of the models consists of gene expression data, copy number alteration, epigenomics, proteomics, and single nucleotide mutations data sets. However, an integrated data analysis strategy combines various omics modalities, and this amalgamation of different types of data could help to develop promising prognostic models. Multi-platform data integration relies on (a) advances in sample extraction and processing technologies; (b) the availability of sufficiently large, matched, and carefully annotated data sets for multi-omics data; (c) molecular and physiologically characterized and graded tumor/cancer data set; (d) data sets with more informative images compared to present databases, e.g., the TCGA image collection (Yu K.-H. et al., 2017) for better 3D-fitting of *in vivo* imaging and *ex vivo* data. The first step in ML analysis is pre-processing of a defined data set(s). It requires normalization, noise filtration, and feature selection when more than one data sets are combined. Normalization becomes an essential step to eliminate biases during the analysis of different data sets that are merged. Selection of defined features is a critical phase in the success of an algorithm in classification, regression, and pattern recognition(Vougas et al., 2019).

#### Selection of Algorithm/Prediction Model and Data Integration

Algorithms are trained through optimizing the parameters to reach an ideal model. k-fold cross-validation (KF-CV) is widely used for optimizing without capturing the noise of data so that the results of statistical analysis can be generalized to an independent data set (Gao et al., 2019). Several studies on statistical methods and algorithms for data integration are reported (Huang et al., 2017; Perakakis et al., 2018; Zeng and Lumley, 2018; Wu C. et al., 2019). Standard machine learning techniques are supervised and unsupervised learning. Supervised learning requires algorithms to be provided with labeled inputs (e.g., omics data) and the desired output (e.g., the presence of a disease or not). In unsupervised learning, data are not labeled, and the algorithm is trained to look for naturally occurring pattern to correspond with the output. Another category that is more common in multi-omics studies is semi-supervised learning, where unlabelled data is used in conjunction with small labeled input. Briefly, multi-omics data integration consists of (a) dimension reduction: to reduce complexity, a number of factors are condensed to fewer variables (called components). (b) Clustering: Grouping input variables with common characteristics in same clusters, (c) density estimation to assess the distribution of input variables in specific space, and (d) regression to estimate the relationships among variables and for developing predictive models.

#### Testing the Prediction Models

Building a model that fits data beyond the current predictive model is the ultimate goal of training a candidate computational model. This can be tested by implementing a candidate predictive model to blind data sets. If the model is for developing tools to identify precision and personalized therapies for individual cancer patients, panels from clinical data sets should be preferentially used. A trained model that fails to generalize might be because of overfitting or underfitting (Dietterich and Bakiri, 1995). In the case of overfitting, noise, or random fluctuations are picked up in the training data, which negatively impact the model’s ability to generalize. Overfitting of a trained model is a major issue in machine learning. In underfitting, the underlying structure of a particular data set is not captured in a set of *in silico* pipeline. The predictive model’s capacity to make predictions understandable or interpretable to humans is another key requirement, i.e., the higher the complexity of the model, the more challenging interpretability becomes (black box models). This could be achieved at different levels of data processing and abstraction, however the development of methods for interpreting ML models is at a relatively early stage, particularly for precision oncology (Castelvecchi, 2016). Enhancing the interpretability will allow users to peer into the hidden layers of the model and determine how exactly the predictions are made on a case-to-case basis.

### Deep Neural Networks: For Multi-Omics Data Integration

Deep neural networks (DNNs) are a subset of machine learning, which is gaining popularity in precision medicine. Today’s complex multi-omics data might be challenging to analyze with traditional machine learning algorithms. DNNs algorithms can integrate multi-omics data with better sensitivity, specificity, and efficiency. Moreover, DNNs have the advantage of integrating other sources of information such as medical images or clinical health records, which is a pre-requisite for personalized medicine. Sakellaropoulos et al. designed the DNNs model, which could capture pathways that linked gene expression with drug response and showed that DNNs are better than other traditional machine learning algorithms. Also, DNNs predicted drug response and survival in a large clinical cohort (Sakellaropoulos et al., 2019). Deep learning is still an emerging area in biomedical field, their effectiveness is not always guaranteed. Cancer multi-omics data integration is done using various approaches: unsupervised cancer subtyping to show patient survival (Ramazzotti et al., 2018), graph-based integration to integrate copy number aberration, epigenome, and transcriptome data sets for ovarian cancer clinical outcome prediction (Kim et al., 2015) and integration DNA methylation and matched imaging data to predict glioblastoma disease progression (Klughammer et al., 2018). However, rigorous mathematical foundations for emerging DNNs architectures are still lacking (Martorell-Marugán et al., 2019). One of the most challenging and futurist modules of the data-integration is combining multi-omics and non-omics data (imaging, biochemical/molecular profile data, clinical symptoms). Yu et al., associated omics data of lung cancer patients with the histopathology data to determine the patient survival rate (Yu K.-H. et al., 2017).

### Machine Learning for Drug Response Prediction in Precision Oncology Applications

Identification of a panel of biomarkers that are associated with treatment responses is imperative for the precision oncology approach. Machine learning algorithms are being developed for prediction to drug response using response-predictive biomarkers through integrative analysis of multi-omics data (Ali and Aittokallio, 2019). Drug sensitivity prediction models, which are entirely based on gene expression profile, are less trustworthy compared to those which are based on integrated multi-omics profiling. Input data type, noise ratio, dimensionality, data complexity, and heterogeneity, are the crucial factors for drug response prediction model development. Sometimes, it is difficult to understand prediction models due to the dominance of gene expression profile data sets, which can be decreased by a two-stage method, called TANDEM (Aben et al., 2016). Bayesian efficient multiple kernel learning (BEMKL) is another drug response prediction model based on multi-omics data. It was the top-performing model in the National Cancer Institute - Dialogue for Reverse Engineering Assessment and Methods (NCI-DREAM7) Drug Sensitivity Prediction Challenge (Costello et al., 2014). Currently, the majority of data in repositories that are publicly available represent a significant set of data that are derived using cell lines treated with different doses of drugs and a large number of compounds. Some of these widely used data sets are: (i) The genomics of Drug Sensitivity in Cancer (GDSC), (ii) Cancer Cell Line Encyclopedia (CCLE), and (iii) National Cancer Institute drug screening panel (NCI-60). It is essential to understand that data extracted from clinical samples are ideal for the development of favorable drug prediction models. Heterogeneous properties of cancers make *in silico* analysis for molecular matching using cancer cell lines challenging in clinical settings (Hanahan and Weinberg, 2011; Turajlic et al., 2019). Importantly, the interplay of tumor-microenvironment that determines cancer development and response to drug treatment cannot be recapitulated using cancer cell lines model, and therefore, molecular changes associated with clinical cancers are diverse than in cancer cell lines (Wu and Dai, 2017). Lack of reliable resources for input data set stalled the success of creating drug prediction models. There is an urgent need to evaluate *in silico* technologies like *transfer learning* (TrLe) methods employing different ML algorithms and applications that utilize predictive feature (very complex non-linear relationships between features) learned in cell line trained model to build a new model or leverage information from auxiliary data not directly belonging to the problem being handled, that can be used in real clinical settings. Several studies have executed TrLe approach and tested and trained machine learning model for data obtained from clinical samples (Daemen et al., 2013; Turki et al., 2018). Turki et al. used TrLe-based approach to transfer patterns learned in breast and lung cancer patient data sets to predict drug sensitivity of multiple myeloma patients (Turki et al., 2018). Daemen et al. used breast cancer cell lines data for training model and tested on clinical data sets derived from TCGA (Daemen et al., 2013). Similarly, the Geeleher group built a training model on gene expression data sets extracted from Cancer Genomics Project and tested them on TCGA data sets from non-small-cell-lung cancers (NSCLC) (Geeleher et al., 2014). Using an elastic net model on to B-cell lymphoma cell lines, Falgreen et al. identified gene signatures that are associated with the development of resistance to drug (cyclophosphamide, doxorubicin, and vincristine) in diffuse large B-cell lymphoma (Falgreen et al., 2015). Sevakula et al. transfer learning for molecular cancer classification using DNN (Sevakula et al., 2019).

### Machine Learning in Biomarker Discovery and Patient Classification

The identification of the disease biomarkers from -omics data does not only facilitate the stratification of patient cohorts but also provides early diagnostic information to improve patient management and prevent adverse outcomes. Coudray et al. applied CNN on whole-slide images obtained from The Cancer Genome Atlas to accurately and automatically classify subtypes of lung cancer, namely adenocarcinoma (LUAD) and squamous cell carcinoma (LUSC) and normal lung tissue (Coudray et al., 2018). Likewise, Huttunen et al., automated classification of multiphoton microscopy images of ovarian tissue (Huttunen et al., 2018). Further, they reported a prediction performance comparable to that obtained by pathologists. Brinker et al., automated dermoscopic melanoma image classification using CNN and showed its superiority over both junior and board-certified dermatologists (Brinker et al., 2019). Molecular profiling of carcinoma using circulating cell-free DNA is another approach for sub-dividing patients in risk factors (Kaseb et al., 2019). It has the advantage of being a noninvasive panel of biomarkers based on the multi-omics approach to increase the accuracy compared to biomarker-based on single omics data. For instance, protein biomarkers found in small sample sizes in the discovery cohort may be prone to achieve over-fitting and overinterpretation of proteomic data. Combined analysis of genomics with proteomics data sets led to the identification of novel therapeutic targets such as altered PI3K pathway in hormone receptor-positive breast cancer (Stemke-Hale et al., 2008). Transcriptomics with proteomics data sets analysis leads to the identification of gonadotropin-releasing hormone (GnRH) signaling pathway in glioblastoma that was not interpreted with single omics data set (Jayaram et al., 2016). Similarly, integrated analysis of DNA copy number alteration, with gene expression data in breast cancer patients led to understand the biology of cancer type and promoted to identify novel therapeutic interventions (Curtis et al., 2012). Four unique urinary biomarkers were identified in an integrated transcriptomic and metabolomics data analysis that was more reliable than single omics data analysis (Nam et al., 2009). Integrated proteogenomic characterization of paired tumor and adjacent liver samples identified alterations of the liver-specific proteome and metabolism. Biomarkers and patients’ subgroups with distinct features in metabolic reprogramming, microenvironment dysregulation, cell proliferation, and potential therapeutics were identified (Gao et al., 2019).

## Concluding Remarks and Outlook

Cancer refers to a compendium of related diseases with uncontrolled dividing and spreading cells. More than 100s of different types of cancers are known. Cancer will be the leading cause of mortality in developed countries by 2030 (Centre for Disease Control). Cancer treatments are challenging due to its heterogenicity (temporal and spatial), high recurrence, and low median survival rate causing millions of deaths every. The molecular understanding of tumor biology has notably changed cancer treatment paradigms during the past 15 years. Still, the success of cancer therapeutics in clinical trials is the lowest of all major diseases. Future cancer treatments thus vouch for tailoring personalized therapies and targeting components of the tumor microenvironment. Accurate early diagnosis and prognosis of cancer greatly increases the chances for successful treatment and patient’s survival rate. Present cancer diagnosis relies on the clinician’s judgment based on their knowledge and clinical experience, which certainly cannot be guaranteed accurate diagnosis. This aspect points to the variability of the human brain to integrate large amounts of sample data. AI (ML and deep learning) is extremely proficient at handling vast amounts of complex nonlinear data (multi-omics and non-omics) generated during cancer treatments and researches, fault tolerance, parallel distributed processing, learning, and decision-making capabilities to improve oncologic care. AI could thus not only integrate various aspects of the clinical diversity but also helps to address the current lack of objectivity and universality in expert systems. Various researches showed impressive diagnostic and prognosis performance of AI using ML (Esteva et al., 2017; Ferroni et al., 2019; Jiang and Xu, 2019). Yoon et al. showed the potential of AI models for personalized oncology treatments that can estimate individualized treatment effects based on the analysis of counterfactual clinical outcomes (Yoon et al., 2018). ML algorithms (supervised or unsupervised learning) guided by clinicians could unravel the hidden molecular patterns within the data sets (multi-omics and non-omics) to support discovery of biomarkers (diagnostic, prognostic, recovery, and recurrence), candidate therapeutic targets associated with a specific patient group, and clinically relevant subtypes without explicit programming in clinical setups. Clinicians’ roles are inevitable in selecting the training data sets and multiple combinations of parameters necessary for building a classification ML model to address specific research questions. In turn, AI can help train junior physicians in clinical diagnosis and decision making. Expanding AI applications from pattern recognition capacity to dealing with multiple data modalities, insufficient data, evaluation of selective and predictive performance, guiding the learning process, and fine-tune models *via* feedback could revolutionize the cancer managements. Another step forward towards AI mediated clinical application is the development of ML pipelines that not only automate the design and evaluation of algorithms but also delineate the clinician the reasoning underlying the model predictions. This is a crucial step considering the fact although AI has learning potential but is in its infancy and cannot be left unattended. Yet another aspect is the extrapolation of the models generated using the cell line data to the patients, as the majority of the previous studies are performed on cell lines or limited small patient sample size, and the portability of the models generated in one cancer to another. AI has come long way but still it must achieve several landmarks: (a) non-reproducible results, (b) population heterogeneity, (c) instrument-variation, (d) lab-to-lab variation, (e) data normalization, (f) cross-compare results by different studies, (g) simulate results *in vitro* to clinics, (h) personalize, and (i) cost-effectiveness. Taken together, advancements in AI-based clinical cancer research will remarkably improve cancer prognosis and diagnosis with precision, resulting in enhanced prediction rates and patient survival.

## Author Contributions

VR and SKP conceived the idea, wrote the manuscript, made table, and figures. BG contributed to writing.

## Conflict of Interest

The authors declare that the research was conducted in the absence of any commercial or financial relationships that could be construed as a potential conflict of interest.

## References

[B1] Abadi S. Yan W. X. Amar D. Mayrose I. (2017). A machine learning approach for predicting CRISPR-Cas9 cleavage efficiencies and patterns underlying its mechanism of action. PLoS Comput. Biol. 13, e1005807. 10.1371/journal.pcbi.1005807 29036168 PMC5658169

[B2] Abbassi-Ghadi N. Golf O. Kumar S. Antonowicz S. McKenzie J. S. Huang J. (2016). Imaging of Esophageal Lymph Node Metastases by Desorption Electrospray Ionization Mass Spectrometry. Cancer Res. 76, 5647–5656. 10.1158/0008-5472.CAN-16-0699 27364550

[B3] Abbatiello S. Ackermann B. L. Borchers C. Bradshaw R. A. Carr S. A. Chalkley R. (2017). New Guidelines for Publication of Manuscripts Describing Development and Application of Targeted Mass Spectrometry Measurements of Peptides and Proteins. Mol. Cell. Proteomics 16, 327–328. 10.1074/mcp.E117.067801 28183812 PMC5340997

[B4] Abdelmoula W. M. Balluff B. Englert S. Dijkstra J. Reinders M. J. T. Walch A. (2016). Data-driven identification of prognostic tumor subpopulations using spatially mapped t-SNE of Mass spectrometry imaging data. Proc. Natl. Acad. Sci. U. S. A. 113, 12244–12249. 10.1073/pnas.1510227113 27791011 PMC5087072

[B5] Aben N. Vis D. J. Michaut M. Wessels L. F. A. (2016). TANDEM: a two-stage approach to maximize interpretability of drug response models based on multiple molecular data types. Bioinformatics 32, i413–i420. 10.1093/bioinformatics/btw449 27587657

[B6] Abu Sammour D. Marsching C. Geisel A. Erich K. Schulz S. Ramallo Guevara C. (2019). Quantitative Mass Spectrometry Imaging Reveals Mutation Status-independent Lack of Imatinib in Liver Metastases of Gastrointestinal Stromal Tumors. Sci. Rep. 9, 10698. 10.1038/s41598-019-47089-5 31337874 PMC6650609

[B7] Addie R. D. Balluff B. Bovée J. V. M. G. Morreau H. McDonnell L. A. (2015). Current State and Future Challenges of Mass Spectrometry Imaging for Clinical Research. Anal. Chem. 87, 6426–6433. 10.1021/acs.analchem.5b00416 25803124

[B8] Alakwaa F. M. Chaudhary K. Garmire L. X. (2018). Deep Learning Accurately Predicts Estrogen Receptor Status in Breast Cancer Metabolomics Data. J. Proteome Res. 17, 337–347. 10.1021/acs.jproteome.7b00595 29110491 PMC5759031

[B9] Ali M. Aittokallio T. (2019). Machine learning and feature selection for drug response prediction in precision oncology applications. Biophys. Rev. 11, 31–39. 10.1007/s12551-018-0446-z 30097794 PMC6381361

[B10] Aliper A. Plis S. Artemov A. Ulloa A. Mamoshina P. Zhavoronkov A. (2016). Deep learning applications for predicting pharmacological properties of drugs and drug repurposing using transcriptomic data. Mol. Pharm. 13, 2524–2530. 10.1021/acs.molpharmaceut.6b00248 27200455 PMC4965264

[B11] Alvarez J. A. Chong E. Y. Walker D. II Chandler J. D. Michalski E. S. Grossmann R. E. (2017). Plasma metabolomics in adults with cystic fibrosis during a pulmonary exacerbation: A pilot randomized study of high-dose vitamin D3 administration. Metabolism 70, 31–41. 10.1016/j.metabol.2017.02.006 28403943 PMC5407388

[B12] Angerer T. B. Magnusson Y. Landberg G. Fletcher J. S. (2016). Lipid heterogeneity resulting from fatty acid processing in the human breast cancer microenvironment identified by GCIB-ToFSIMS imaging. Anal. Chem. 88, 11946–11954. 10.1021/acs.analchem.6b03884 27783898

[B13] Awasthi G. Tyagi S. Kumar V. Patel S. K. Rojh D. Sakrappanavar V. (2018). A Proteogenomic Analysis of Haptoglobin in Malaria. Proteomics - Clin. Appl. 12, e1700077. 10.1002/prca.201700077 28960920

[B14] Azuaje F. Kim S.-Y. Perez Hernandez D. Dittmar G. (2019). Connecting Histopathology Imaging and Proteomics in Kidney Cancer through Machine Learning. J. Clin. Med. 8:1535. 10.3390/jcm8101535 PMC6832975 31557788

[B15] Balog J. Sasi-Szabó L. Kinross J. Lewis M. R. Muirhead L. J. Veselkov K. (2013). Intraoperative tissue identification using rapid evaporative ionization mass spectrometry. Sci. Transl. Med. 5, 194ra93. 10.1126/scitranslmed.3005623 23863833

[B16] Bedi G. Carrillo F. Cecchi G. A. Slezak D. F. Sigman M. Mota N. B. (2015). Automated analysis of free speech predicts psychosis onset in high-risk youths. NPJ Schizophr. 1, 1–7. 10.1038/npjschz.2015.30 PMC4849456 27336038

[B17] Bednarczyk K. Gawin M. Chekan M. Kurczyk A. Mrukwa G. Pietrowska M. (2019). Discrimination of normal oral mucosa from oral cancer by mass spectrometry imaging of proteins and lipids. J. Mol. Histol. 50, 1–10. 10.1007/s10735-018-9802-3 30390197 PMC6323087

[B18] Bejnordi B. E. Veta M. Van Diest P. J. Van Ginneken B. Karssemeijer N. Litjens G. (2017). Diagnostic assessment of deep learning algorithms for detection of lymph node metastases in women with breast cancer. JAMA - J. Am. Med. Assoc. 318, 2199–2210. 10.1001/jama.2017.14585 PMC5820737 29234806

[B19] Bertini I. Calabró A. De Carli V. Luchinat C. Nepi S. Porfirio B. (2009). The metabonomic signature of celiac disease. J. Proteome Res. 8, 170–177. 10.1021/pr800548z 19072164

[B20] Bhasin J. M. Lee B. H. Matkin L. Taylor M. G. Hu B. Xu Y. (2015). Methylome-wide Sequencing Detects DNA Hypermethylation Distinguishing Indolent from Aggressive Prostate Cancer. Cell Rep. 13, 2135–2146. 10.1016/j.celrep.2015.10.078 26628371 PMC4684962

[B21] Bi W. L. Hosny A. Schabath M. B. Giger M. L. Birkbak N. J. Mehrtash A. (2019). Artificial intelligence in cancer imaging: Clinical challenges and applications. CA. Cancer J. Clin 69, 127-157. 10.3322/caac.21552 30720861 PMC6403009

[B22] Biglarian A. Hajizadeh E. Kazemnejad A. Zali M. R. (2011). Application of artificial neural network in predicting the survival rate of gastric cancer patients. Iran. J. Public Health 40, 80–86. PMC3481773 23113076

[B23] Bottaci L. Drew P. J. Hartley J. E. Hadfield M. B. Farouk R. Lee P. W. (1997). Artificial neural networks applied to outcome prediction for colorectal cancer patients in separate institutions. Lancet (Lond. Engl.) 350, 469–472. 10.1016/S0140-6736(96)11196-X 9274582

[B24] Brat D. J. Verhaak R. G. W. Aldape K. D. Yung W. K. A. Salama S. R. Cooper L. A. D. (2015). Comprehensive, integrative genomic analysis of diffuse lower-grade gliomas. N. Engl. J. Med. 372, 2481–2498. 10.1056/NEJMoa1402121 26061751 PMC4530011

[B25] Briggs M. T. Condina M. R. Ho Y. Y. Everest-Dass A. V. Mittal P. Kaur G. (2019). MALDI Mass Spectrometry Imaging of Early- and Late-Stage Serous Ovarian Cancer Tissue Reveals Stage-Specific N-Glycans. Proteomics 19, e1800482. 10.1002/pmic.201800482 31364262

[B26] Brinker T. J. Hekler A. Enk A. H. Berking C. Haferkamp S. Hauschild A. (2019). Deep neural networks are superior to dermatologists in melanoma image classification. Eur. J. Cancer 119, 11–17. 10.1016/j.ejca.2019.05.023 31401469

[B27] Buchhalter I. Hutter B. Alioto T. S. Beck T. A. Boutros P. C. Brors B. (2014). A comprehensive multicenter comparison of whole genome sequencing pipelines using a uniform tumor-normal sample pair. Cold Spring Harbor Labs J. 013177. 10.1101/013177

[B28] Calligaris D. Feldman D. R. Norton I. Brastianos P. K. Dunn I. F. Santagata S. (2015). Molecular typing of meningiomas by desorption electrospray ionization mass spectrometry imaging for surgical decision-making. Int. J. Mass Spectrom. 377, 690–698. 10.1016/j.ijms.2014.06.024 25844057 PMC4379512

[B29] Cancer Moonshot - National Cancer Institute (2016). Natl. Cancer Inst. Available at: https://www.cancer.gov/research/key-initiatives/moonshot-cancer-initiative (Accessed April 19, 2020).

[B30] Carter C. L. Jones J. W. Farese A. M. MacVittie T. J. Kane M. A. (2017). Lipidomic dysregulation within the lung parenchyma following whole-thorax lung irradiation: Markers of injury, inflammation and fibrosis detected by MALDI-MSI. Sci. Rep. 7, 10343. 10.1038/s41598-017-10396-w 28871103 PMC5583385

[B31] Castelvecchi D. (2016). Can we open the black box of AI? Nature 538, 20–23. 10.1038/538020a 27708329

[B32] cBioPortal for Cancer Genomics Available at: http://www.cbioportal.org/ (Accessed March 24, 2020).

[B33] Cerami E. Gao J. Dogrusoz U. Gross B. E. Sumer S. O. Aksoy B. A. (2012). The cBio Cancer Genomics Portal: An open platform for exploring multidimensional cancer genomics data. Cancer Discov. 2, 401–404. 10.1158/2159-8290.CD-12-0095 22588877 PMC3956037

[B34] Chakraborty S. Hosen M. II Ahmed M. Shekhar H. U. (2018). Onco-Multi-OMICS Approach: A New Frontier in Cancer Research. BioMed. Res. Int. 2018:9836256. 10.1155/2018/9836256 30402498 PMC6192166

[B35] Chang S.-W. Kareem S. A. Kallarakkal T. G. Merican A. F. M. A. Abraham M. T. Zain R. B. (2011). Feature selection methods for optimizing clinicopathologic input variables in oral cancer prognosis. Asian Pac. J. Cancer Prev. 12, 2659–2664. 22320970

[B36] Chang E. K. Yu C. Y. Clarke R. Hackbarth A. Sanders T. Esrailian E. (2016). Defining a Patient Population With Cirrhosis. J. Clin. Gastroenterol. 50, 889–894. 10.1097/MCG.0000000000000583 27348317

[B37] Chang K. Bai H. X. Zhou H. Su C. Bi W. L. Agbodza E. (2018). Residual convolutional neural network for the determination of IDH status in low- and high-grade gliomas from mr imaging. Clin. Cancer Res. 24, 1073–1081. 10.1158/1078-0432.CCR-17-2236 29167275 PMC6051535

[B38] Chaudhary K. Poirion O. B. Lu L. Garmire L. X. (2018). Deep Learning-Based Multi-Omics Integration Robustly Predicts Survival in Liver Cancer. Clin. Cancer Res. 24, 1248–1259. 10.1158/1078-0432.CCR-17-0853 28982688 PMC6050171

[B39] Chorell E. Hall U. A. Gustavsson C. Berntorp K. Puhkala J. Luoto R. (2017). Pregnancy to postpartum transition of serum metabolites in women with gestational diabetes. Metabolism 72, 27–36. 10.1016/j.metabol.2016.12.018 28641781

[B40] Chughtai K. Jiang L. Greenwood T. R. Glunde K. Heeren R. M. A. (2013). Mass spectrometry images acylcarnitines, phosphatidylcholines, and sphingomyelin in MDA-MB-231 breast tumor models. J. Lipid Res. 54, 333–344. 10.1194/jlr.M027961 22930811 PMC3588863

[B41] Clark A. R. Calligaris D. Regan M. S. Pomeranz Krummel D. Agar J. N. Kallay L. (2018). Rapid discrimination of pediatric brain tumors by mass spectrometry imaging. J. Neurooncol. 140, 269–279. 10.1007/s11060-018-2978-2 30128689 PMC6244779

[B42] Cohen J. D. Li L. Wang Y. Thoburn C. Afsari B. Danilova L. (2018). Detection and localization of surgically resectable cancers with a multi-analyte blood test. Sci. (80-. ). 359, 926–930. 10.1126/science.aar3247 PMC6080308 29348365

[B43] Cornett D. S. Mobley J. A. Dias E. C. Andersson M. Arteaga C. L. Sanders M. E. (2006). A novel histology-directed strategy for MALDI-MS tissue profiling that improves throughput and cellular specificity in human breast cancer. Mol. Cell. Proteomics 5, 1975–1983. 10.1074/mcp.M600119-MCP200 16849436

[B44] Costello J. C. Heiser L. M. Georgii E. Gönen M. Menden M. P. Wang N. J. (2014). A community effort to assess and improve drug sensitivity prediction algorithms. Nat. Biotechnol. 32, 1202–1212. 10.1038/nbt.2877 24880487 PMC4547623

[B45] Coudray N. Ocampo P. S. Sakellaropoulos T. Narula N. Snuderl M. Fenyö D. (2018). Classification and mutation prediction from non-small cell lung cancer histopathology images using deep learning. Nat. Med. 24, 1559–1567. 10.1038/s41591-018-0177-5 30224757 PMC9847512

[B46] Cui M. Wang Q. Chen G. (2016). Serum metabolomics analysis reveals changes in signaling lipids in breast cancer patients. Biomed. Chromatogr. 30, 42–47. 10.1002/bmc.3556 26179699

[B47] Curtis C. Shah S. P. Chin S. F. Turashvili G. Rueda O. M. Dunning M. J. (2012). The genomic and transcriptomic architecture of 2,000 breast tumours reveals novel subgroups. Nature 486, 346–352. 10.1038/nature10983 22522925 PMC3440846

[B48] Daemen A. Griffith O. L. Heiser L. M. Wang N. J. Enache O. M. Sanborn Z. (2013). Modeling precision treatment of breast cancer. Genome Biol. 14, R110. 10.1186/gb-2013-14-10-r110 24176112 PMC3937590

[B49] Delcourt V. Franck J. Leblanc E. Narducci F. Robin Y. M. Gimeno J. P. (2017). Combined Mass Spectrometry Imaging and Top-down Microproteomics Reveals Evidence of a Hidden Proteome in Ovarian Cancer. EBioMedicine 21, 55–64. 10.1016/j.ebiom.2017.06.001 28629911 PMC5514399

[B50] Delen D. Walker G. Kadam A. (2005). Predicting breast cancer survivability: A comparison of three data mining methods. Artif. Intell. Med. 34, 113–127. 10.1016/j.artmed.2004.07.002 15894176

[B51] Dietterich T. G. Bakiri G. (1995). Solving Multiclass Learning Problems via Error-Correcting Output Codes (AI Access Foundation and Morgan Kaufmann Publishers).

[B52] Doerr A. (2019). Single-cell proteomics. Nat. Methods 16, 20. 10.1038/s41592-018-0273-y 30573843

[B53] Dória M. L. McKenzie J. S. Mroz A. Phelps D. L. Speller A. Rosini F. (2016). Epithelial ovarian carcinoma diagnosis by desorption electrospray ionization mass spectrometry imaging. Sci. Rep. 6. 10.1038/srep39219 PMC5156945 27976698

[B54] Duan K. B. Rajapakse J. C. Wang H. Azuaje F. (2005). Multiple SVM-RFE for gene selection in cancer classification with expression data. IEEE Trans. Nanobiosci. 4, 228–233. 10.1109/TNB.2005.853657 16220686

[B55] Eisner R. Greiner R. Tso V. Wang H. Fedorak R. N. (2013). A Machine-Learned Predictor of Colonic Polyps Based on Urinary Metabolomics. BioMed. Res. Int. 2013, 11. 10.1155/2013/303982 PMC3838851 24307992

[B56] Esteva A. Kuprel B. Novoa R. A. Ko J. Swetter S. M. Blau H. M. (2017). Dermatologist-level classification of skin cancer with deep neural networks. Nature 542, 115–118. 10.1038/nature21056 28117445 PMC8382232

[B57] Eun B. Sampley M. L. Good A. L. Gebert C. M. Pfeifer K. (2013). Promoter cross-talk via a shared enhancer explains paternally biased expression of Nctc1 at the Igf2/H19/Nctc1 imprinted locus. Nucleic Acids Res. 41, 817–826. 10.1093/nar/gks1182 23221643 PMC3553941

[B58] Falgreen S. Dybkær K. Young K. H. Xu-Monette Z. Y. El-Galaly T. C. Laursen M. B. (2015). Predicting response to multidrug regimens in cancer patients using cell line experiments and regularised regression models. BMC Cancer 15, 235. 10.1186/s12885-015-1237-6 25881228 PMC4396063

[B59] Fayazfar S. Zali H. Arefi Oskouie A. Asadzadeh Aghdaei H. Rezaei Tavirani M. Nazemalhosseini Mojarad E. (2019). Early diagnosis of colorectal cancer via plasma proteomic analysis of CRC and advanced adenomatous polyp. Gastroenterol. Hepatol. Bed Bench 12, 328–339. 31749922 PMC6820836

[B60] Ferroni P. Zanzotto F. M. Riondino S. Scarpato N. Guadagni F. Roselli M. (2019). Breast cancer prognosis using a machine learning approach. Cancers (Basel). 11. 10.3390/cancers11030328 PMC6468737 30866535

[B61] Forbes S. A. Beare D. Gunasekaran P. Leung K. Bindal N. Boutselakis H. (2015). COSMIC: exploring the world’s knowledge of somatic mutations in human cancer. Nucleic Acids Res. 43, D805–D811. 10.1093/nar/gku1075 25355519 PMC4383913

[B62] Gao J. Aksoy B. A. Dogrusoz U. Dresdner G. Gross B. Sumer S. O. (2013). Integrative analysis of complex cancer genomics and clinical profiles using the cBioPortal. Sci. Signal. 6, pl1. 10.1126/scisignal.2004088 23550210 PMC4160307

[B63] Gao Q. Zhu H. Dong L. Shi W. Chen R. Song Z. (2019). Integrated Proteogenomic Characterization of HBV-Related Hepatocellular Carcinoma. Cell 179, 561–577.e22. 10.1016/j.cell.2019.08.052 31585088

[B64] Garvin J. H. Kim Y. Gobbel G. T. Matheny M. E. Redd A. Bray B. E. (2018). Automating quality measures for heart failure using natural language processing:a descriptive study in the department of veterans affairs. J. Med. Internet Res. 20, e5. 10.2196/medinform.9150 29335238 PMC5789165

[B65] Geeleher P. Cox N. J. Huang R. S. (2014). Clinical drug response can be predicted using baseline gene expression levels and in vitro drug sensitivity in cell lines. Genome Biol. 15, R47. 10.1186/gb-2014-15-3-r47 24580837 PMC4054092

[B66] Goldman M. Craft B. Swatloski T. Ellrott K. Cline M. Diekhans M. (2013). The UCSC Cancer Genomics Browser: update 2013. Nucleic Acids Res. 41, D949–D954. 10.1093/nar/gks1008 23109555 PMC3531186

[B67] Gonzalez-Perez A. Perez-Llamas C. Deu-Pons J. Tamborero D. Schroeder M. P. Jene-Sanz A. (2013). IntOGen-mutations identifies cancer drivers across tumor types. Nat. Methods 10, 1081–1082. 10.1038/nmeth.2642 24037244 PMC5758042

[B68] Guan W. Zhou M. Hampton C. Y. Benigno B. B. Walker L. D. E. Gray A. (2009). Ovarian cancer detection from metabolomic liquid chromatography/mass spectrometry data by support vector machines. BMC Bioinf. 10:259. 10.1186/1471-2105-10-259 PMC2741455 19698113

[B69] Guenther S. Muirhead L. J. Speller A. V. M. Golf O. Strittmatter N. Ramakrishnan R. (2015). Spatially resolved metabolic phenotyping of breast cancer by desorption electrospray ionization mass spectrometry. Cancer Res. 75, 1828–1837. 10.1158/0008-5472.CAN-14-2258 25691458

[B70] Guo S. Wang Y. Zhou D. Li Z. (2014). Significantly increased monounsaturated lipids relative to polyunsaturated lipids in six types of cancer microenvironment are observed by mass spectrometry imaging. Sci. Rep. 4, 5959. 10.1038/srep05959 25091112 PMC4121604

[B71] Haenssle H. A. Fink C. Schneiderbauer R. Toberer F. Buhl T. Blum A. (2018). Man against machine: diagnostic performance of a deep learning convolutional neural network for dermoscopic melanoma recognition in comparison to 58 dermatologists. Ann. Oncol. 29, 1836–1842. 10.1093/annonc/mdy166 29846502

[B72] Hanahan D. Weinberg R. A. (2011). Hallmarks of cancer: The next generation. Cell 144, 646–674. 10.1016/j.cell.2011.02.013 21376230

[B73] Hasin Y. Seldin M. Lusis A. (2017). Multi-omics approaches to disease. Genome Biol. 18, 1–15. 10.1186/s13059-017-1215-1 28476144 PMC5418815

[B74] Hasnain Z. Mason J. Gill K. Miranda G. Gill I. S. Kuhn P. (2019). Machine learning models for predicting post-cystectomy recurrence and survival in bladder cancer patients. PLoS One 14, e0210976. 10.1371/journal.pone.0210976 30785915 PMC6382101

[B75] He X. Chang S. Zhang J. Zhao Q. Xiang H. Kusonmano K. (2008). MethyCancer: The database of human DNA methylation and cancer. Nucleic Acids Res. 36, D836–D841. 10.1093/nar/gkm730 17890243 PMC2238864

[B76] Heinemann J. Mazurie A. Tokmina-Lukaszewska M. Beilman G. J. Bothner B. (2014). Application of support vector machines to metabolomics experiments with limited replicates. Metabolomics 10, 1121–1128. 10.1007/s11306-014-0651-0

[B77] Hinsch A. Buchholz M. Odinga S. Borkowski C. Koop C. Izbicki J. R. (2017). MALDI imaging mass spectrometry reveals multiple clinically relevant masses in colorectal cancer using large-scale tissue microarrays. J. Mass Spectrom. 52, 165–173. 10.1002/jms.3916 28117928

[B78] Hiraide T. Ikegami K. Sakaguchi T. Morita Y. Hayasaka T. Masaki N. (2016). Accumulation of arachidonic acid-containing phosphatidylinositol at the outer edge of colorectal cancer. Sci. Rep. 6, 29935. 10.1038/srep29935 27435310 PMC4951683

[B79] Holzlechner M. Bonta M. Lohninger H. Limbeck A. Marchetti-Deschmann M. (2018). Multisensor Imaging-From Sample Preparation to Integrated Multimodal Interpretation of LA-ICPMS and MALDI MS Imaging Data. Anal. Chem. 90, 8831–8837. 10.1021/acs.analchem.8b00816 29961333

[B80] Huang S. Chaudhary K. Garmire L. X. (2017). More is better: Recent progress in multi-omics data integration methods. Front. Genet. 8, 84. 10.3389/fgene.2017.00084 28670325 PMC5472696

[B81] Huang L. Mao X. Sun C. Luo Z. Song X. Li X. (2019). A graphical data processing pipeline for mass spectrometry imaging-based spatially resolved metabolomics on tumor heterogeneity. Anal. Chim. Acta 1077, 183–190. 10.1016/j.aca.2019.05.068 31307708

[B82] Huang S. Yang J. Fong S. Zhao Q. (2020). Artificial intelligence in cancer diagnosis and prognosis: Opportunities and challenges. Cancer Lett. 471, 61–71. 10.1016/j.canlet.2019.12.007 31830558

[B83] Huber K. Feuchtinger A. Borgmann D. M. Li Z. Aichler M. Hauck S. M. (2014). Novel approach of MALDI drug imaging, immunohistochemistry, and digital image analysis for drug distribution studies in tissues. Anal. Chem. 86, 10568–10575. 10.1021/ac502177y 25263480

[B84] Huttunen M. J. Hassan A. McCloskey C. W. Fasih S. Upham J. Vanderhyden B. C. (2018). Automated classification of multiphoton microscopy images of ovarian tissue using deep learning. J. Biomed. Opt. 23, 1. 10.1117/1.jbo.23.6.066002 29900705

[B85] Ibrahim A. Gamble P. Jaroensri R. Abdelsamea M. M. Mermel C. H. Chen P.-H. C. (2020). Artificial intelligence in digital breast pathology: Techniques and applications. Breast 49, 267–273. 10.1016/j.breast.2019.12.007 31935669 PMC7375550

[B86] Ikeda A. Nishiumi S. Shinohara M. Yoshie T. Hatano N. Okuno T. (2012). Serum metabolomics as a novel diagnostic approach for gastrointestinal cancer. Biomed. Chromatogr. 26, 548–558. 10.1002/bmc.1671 21773981

[B87] Inglese P. McKenzie J. S. Mroz A. Kinross J. Veselkov K. Holmes E. (2017). Deep learning and 3D-DESI imaging reveal the hidden metabolic heterogeneity of cancer††Electronic supplementary information (ESI) available. See DOI: 10.1039/c6sc03738kClick here for additional data file.Click here for additional data file.Click here for. Chem. Sci. 8, 3500–3511. 10.1039/c6sc03738k 28507724 PMC5418631

[B88] Jarmusch A. K. Alfaro C. M. Pirro V. Hattab E. M. Cohen-Gadol A. A. Cooks R. G. (2016). Differential Lipid Profiles of Normal Human Brain Matter and Gliomas by Positive and Negative Mode Desorption Electrospray Ionization - Mass Spectrometry Imaging. PLoS One 11, e0163180. 10.1371/journal.pone.0163180 27658243 PMC5033406

[B89] Jayaram S. Gupta M. K. Raju R. Gautam P. Sirdeshmukh R. (2016). Multi-Omics Data Integration and Mapping of Altered Kinases to Pathways Reveal Gonadotropin Hormone Signaling in Glioblastoma. OMICS 20, 736–746. 10.1089/omi.2016.0142 27930095

[B90] Jiang N. Xu X. (2019). Exploring the survival prognosis of lung adenocarcinoma based on the cancer genome atlas database using artificial neural network. Med. (Baltimore). 98, e15642. 10.1097/MD.0000000000015642 PMC6531113 31096483

[B91] Jiang L. Chughtai K. Purvine S. O. Bhujwalla Z. M. Raman V. Paša-Tolić L. (2015). MALDI-Mass Spectrometric Imaging Revealing Hypoxia-Driven Lipids and Proteins in a Breast Tumor Model. Anal. Chem. 87, 5947–5956. 10.1021/ac504503x 25993305 PMC4820759

[B92] Jiang F. Jiang Y. Zhi H. Dong Y. Li H. Ma S. (2017). Artificial intelligence in healthcare: past, present and future. Stroke Vasc. Neurol. 2, 230–243. 10.1136/svn-2017-000101 29507784 PMC5829945

[B93] Jović S. Miljković M. Ivanović M. Šaranović M. Arsić M. (2017). Prostate Cancer Probability Prediction By Machine Learning Technique. Cancer Invest. 35, 647–651. 10.1080/07357907.2017.1406496 29243988

[B94] Karsan A. Eigl B. J. Flibotte S. Gelmon K. Switzer P. Hassell P. (2005). Analytical and preanalytical biases in serum proteomic pattern analysis for breast cancer diagnosis. Clin. Chem. 51, 1525–1528. 10.1373/clinchem.2005.050708 15951319

[B95] Kaseb A. O. Sánchez N. S. Sen S. Kelley R. K. Tan B. Bocobo A. G. (2019). Molecular Profiling of Hepatocellular Carcinoma Using Circulating Cell-Free DNA. Clin. Cancer Res. 25, 6107–6118. 10.1158/1078-0432.CCR1-18-3341 31363003 PMC9292132

[B96] Kelley D. Z. Flam E. L. Izumchenko E. Danilova L. V. Wulf H. A. Guo T. (2017). Integrated analysis of whole-genome ChIP-Seq and RNA-Seq data of primary head and neck tumor samples associates HPV integration sites with open chromatin marks. Cancer Res. 77, 6538–6550. 10.1158/0008-5472.CAN-17-0833 28947419 PMC6029614

[B97] Kelly R. S. Heiden M. G. V. Giovannucci E. Mucci L. A. (2016). Metabolomic biomarkers of prostate cancer: Prediction, diagnosis, progression, prognosis, and recurrence. Cancer Epidemiol. Biomarkers Prev. 25, 887–906. 10.1158/1055-9965.EPI-15-1223 27197278 PMC4891222

[B98] Kim D. Joung J.-G. Sohn K.-A. Shin H. Park Y. R. Ritchie M. D. (2015). Knowledge boosting: a graph-based integration approach with multi-omics data and genomic knowledge for cancer clinical outcome prediction. J. Am. Med. Inform. Assoc. 22, 109–120. 10.1136/amiajnl-2013-002481 25002459 PMC4433357

[B99] Klughammer J. Kiesel B. Roetzer T. Fortelny N. Nemc A. Nenning K.-H. (2018). The DNA methylation landscape of glioblastoma disease progression shows extensive heterogeneity in time and space. Nat. Med. 24, 1611–1624. 10.1038/s41591-018-0156-x 30150718 PMC6181207

[B100] Korbar B. Olofson A. Miraflor A. Nicka C. Suriawinata M. Torresani L. (2017). Deep learning for classification of colorectal polyps on whole-slide images. J. Pathol. Inform. 8, 30. 10.4103/jpi.jpi_34_17 28828201 PMC5545773

[B101] Koutros S. Meyer T. E. Fox S. D. Issaq H. J. Veenstra T. D. Huang W.-Y. (2013). Prospective evaluation of serum sarcosine and risk of prostate cancer in the Prostate, Lung, Colorectal and Ovarian Cancer Screening Trial. Carcinogenesis 34, 2281–2285. 10.1093/carcin/bgt176 23698636 PMC3786375

[B102] Kriegsmann M. Casadonte R. Kriegsmann J. Dienemann H. Schirmacher P. Hendrik Kobarg J. (2016). Reliable Entity Subtyping in Non-small Cell Lung Cancer by Matrix-assisted Laser Desorption/Ionization Imaging Mass Spectrometry on Formalin-fixed Paraffin-embedded Tissue Specimens. Mol. Cell. Proteomics 15, 3081–3089. 10.1074/mcp.M115.057513 27473201 PMC5054336

[B103] Kunzke T. Balluff B. Feuchtinger A. Buck A. Langer R. Luber B. (2017). Native glycan fragments detected by MALDI-FT-ICR mass spectrometry imaging impact gastric cancer biology and patient outcome. Oncotarget 8, 68012–68025. 10.18632/oncotarget.19137 28978092 PMC5620232

[B104] Kuo R.-J. Huang M.-H. Cheng W.-C. Lin C.-C. Wu Y.-H. (2015). Application of a two-stage fuzzy neural network to a prostate cancer prognosis system. Artif. Intell. Med. 63, 119–133. 10.1016/j.artmed.2014.12.008 25576196

[B105] Lai C.-H. Chang N.-W. Lin C.-F. Lin C.-D. Lin Y.-J. Wan L. (2010). Proteomics-based identification of haptoglobin as a novel plasma biomarker in oral squamous cell carcinoma. Clin. Chim. Acta 411, 984–991. 10.1016/j.cca.2010.03.028 20359475

[B106] Ley T. J. Mardis E. R. Ding L. Fulton B. McLellan M. D. Chen K. (2008). DNA sequencing of a cytogenetically normal acute myeloid leukaemia genome. Nature 456, 66–72. 10.1038/nature07485 18987736 PMC2603574

[B107] Li Y. Song X. Zhao X. Zou L. Xu G. (2014). Serum metabolic profiling study of lung cancer using ultra high performance liquid chromatography/quadrupole time-of-flight mass spectrometry. J. Chromatogr. B Anal. Technol. Biomed. Life Sci. 966, 147–153. 10.1016/j.jchromb.2014.04.047 24856296

[B108] Li Q. Freeman L. M. Rush J. E. Huggins G. S. Kennedy A. D. Labuda J. A. (2015). Veterinary Medicine and Multi-Omics Research for Future Nutrition Targets: Metabolomics and Transcriptomics of the Common Degenerative Mitral Valve Disease in Dogs. OMICS 19, 461–470. 10.1089/omi.2015.0057 26154239

[B109] Li T. He J. Mao X. Bi Y. Luo Z. Guo C. (2015). In situ biomarker discovery and label-free molecular histopathological diagnosis of lung cancer by ambient mass spectrometry imaging. Sci. Rep. 5. 10.1038/srep14089 PMC4585892 26404114

[B110] Li Y. Wu F. X. Ngom A. (2018). A review on machine learning principles for multi-view biological data integration. Brief. Bioinform. 19, 325–340. 10.1093/bib/bbw113 28011753

[B111] Li H. Siddiqui O. Zhang H. Guan Y. (2019). Joint learning improves protein abundance prediction in cancers. BMC Biol. 17, 107. 10.1186/s12915-019-0730-9 31870366 PMC6929375

[B112] Li X. Zhang S. Zhang Q. Wei X. Pan Y. Zhao J. (2019). Diagnosis of thyroid cancer using deep convolutional neural network models applied to sonographic images: a retrospective, multicohort, diagnostic study. Lancet Oncol. 20, 193–201. 10.1016/S1470-2045(18)30762-9 30583848 PMC7083202

[B113] Li L. Zheng X. Zhou Q. Villanueva N. Nian W. Liu X. (2020). Metabolomics-Based Discovery of Molecular Signatures for Triple Negative Breast Cancer in Asian Female Population. Sci. Rep. 10, 370. 10.1038/s41598-019-57068-5 31941951 PMC6962155

[B114] Liang C. A. Chen L. Wahed A. Nguyen A. N. D. (2019). Proteomics analysis of FLT3-ITD mutation in acute myeloid leukemia using deep learning neural network. Ann. Clin. Lab. Sci. 49, 119–126. 10.1093/ajcp/aqx121.148 30814087

[B115] Liebl H. (1967). Ion microprobe mass analyzer. J. Appl. Phys. 38, 5277–5283. 10.1063/1.1709314

[B116] Lin X. Wang Q. Yin P. Tang L. Tan Y. Li H. (2011). A method for handling metabonomics data from liquid chromatography/mass spectrometry: Combinational use of support vector machine recursive feature elimination, genetic algorithm and random forest for feature selection. Metabolomics 7, 549–558. 10.1007/s11306-011-0274-7

[B117] Liu Y. Gonzàlez-Porta M. Santos S. Brazma A. Marioni J. C. Aebersold R. (2017). Impact of Alternative Splicing on the Human Proteome. Cell Rep. 20, 1229–1241. 10.1016/j.celrep.2017.07.025 28768205 PMC5554779

[B118] López de Maturana E. Alonso L. Alarcón P. Martín-Antoniano I. A. Pineda S. Piorno L. (2019). Challenges in the Integration of Omics and Non-Omics Data. Genes (Basel). 10. 10.3390/genes10030238 PMC6471713 30897838

[B119] Lou S. Balluff B. Cleven A. H. G. Bovée J. V. M. G. McDonnell L. A. (2017). Prognostic Metabolite Biomarkers for Soft Tissue Sarcomas Discovered by Mass Spectrometry Imaging. J. Am. Soc Mass Spectrom. 28, 376–383. 10.1007/s13361-016-1544-4 27873216 PMC5227002

[B120] Low S.-K. Zembutsu H. Nakamura Y. (2018). Breast cancer: The translation of big genomic data to cancer precision medicine. Cancer Sci. 109, 497–506. 10.1111/cas.13463 29215763 PMC5834810

[B121] Lu C. F. Hsu F. T. Hsieh K. L. C. Kao Y. C. J. Cheng S. J. Hsu J. B. K. (2018). Machine learning–based radiomics for molecular subtyping of gliomas. Clin. Cancer Res. 24, 4429–4436. 10.1158/1078-0432.CCR-17-3445 29789422

[B122] Lu Y. Yu Q. Gao Y. Zhou Y. Liu G. Dong Q. (2018). Identification of metastatic lymph nodes in MR imaging with faster region-based convolutional neural networks. Cancer Res. 78, 5135–5143. 10.1158/0008-5472.CAN-18-0494 30026330

[B123] Lu T.-P. Kuo K.-T. Chen C.-H. Chang M.-C. Lin H.-P. Hu Y.-H. (2019). Developing a Prognostic Gene Panel of Epithelial Ovarian Cancer Patients by a Machine Learning Model. Cancers (Basel). 11. 10.3390/cancers11020270 PMC6406249 30823599

[B124] Lynch C. M. Abdollahi B. Fuqua J. D. de Carlo A. R. Bartholomai J. A. Balgemann R. N. (2017). Prediction of lung cancer patient survival via supervised machine learning classification techniques. Int. J. Med. Inform. 108, 1–8. 10.1016/j.ijmedinf.2017.09.013 29132615 PMC5726571

[B125] Ma Y. Zhang P. Wang F. Liu W. Yang J. Qin H. (2012). An integrated proteomics and metabolomics approach for defining oncofetal biomarkers in the colorectal cancer. Ann. Surg. 255, 720–730. 10.1097/SLA.0b013e31824a9a8b 22395091

[B126] Maes E. Cho W. C. Baggerman G. (2015). Translating clinical proteomics: the importance of study design. Expert Rev. Proteomics 12, 217–219. 10.1586/14789450.2015.1041512 25925311

[B127] Mallavarapu T. Hao J. Kim Y. Oh J. H. Kang M. (2019). Pathway-based deep clustering for molecular subtyping of cancer. Methods 173, 24–31. 10.1016/j.ymeth.2019.06.017 31247294 PMC7378959

[B128] Manna S. K. Tanaka N. Krausz K. W. Haznadar M. Xue X. Matsubara T. (2014). Biomarkers of coordinate metabolic reprogramming in colorectal tumors in mice and humans. Gastroenterology 146, 1313–1324. 10.1053/j.gastro.2014.01.017 24440673 PMC3992178

[B129] Margulis K. Chiou A. S. Aasi S. Z. Tibshirani R. J. Tang J. Y. Zare R. N. (2018). Distinguishing malignant from benign microscopic skin lesions using desorption electrospray ionization mass spectrometry imaging. Proc. Natl. Acad. Sci. U. S. A. 115, 6347–6352. 10.1073/pnas.1803733115 29866838 PMC6016785

[B130] Martínez-Bartolomé S. Binz P.-A. Albar J. P. (2014). The Minimal Information about a Proteomics Experiment (MIAPE) from the Proteomics Standards Initiative. Methods Mol. Biol. 1072, 765–780. 10.1007/978-1-62703-631-3_53 24136562

[B131] Martorell-Marugán J. Tabik S. Benhammou Y. del Val C. Zwir I. Herrera F. (2019). "Deep Learning in Omics Data Analysis and Precision Medicine," in *Computational Biology* Ed. Husi H. (Brisbane (AU): Codon Publications). 31815397

[B132] Mertins P. Mani D. R. Ruggles K. V. Gillette M. A. Clauser K. R. Wang P. (2016). Proteogenomics connects somatic mutations to signalling in breast cancer. Nature 534, 55–62. 10.1038/nature18003 27251275 PMC5102256

[B133] Miotto R. Li L. Kidd B. A. Dudley J. T. (2016). Deep Patient: An Unsupervised Representation to Predict the Future of Patients from the Electronic Health Records. Sci. Rep. 6, 1–10. 10.1038/srep26094 27185194 PMC4869115

[B134] Mishra S. Srivastava A. K. Suman S. Kumar V. Shukla Y. (2015). Circulating miRNAs revealed as surrogate molecular signatures for the early detection of breast cancer. Cancer Lett. 369, 67–75. 10.1016/j.canlet.2015.07.045 26276721

[B135] Moarii M. Boeva V. Vert J.-P. Reyal F. (2015). Changes in correlation between promoter methylation and gene expression in cancer. BMC Genomics 16, 873. 10.1186/s12864-015-1994-2 26510534 PMC4625954

[B136] Mondul A. M. Moore S. C. Weinstein S. J. Männistö S. Sampson J. N. Albanes D. (2014). 1-Stearoylglycerol is associated with risk of prostate cancer: Results from a serum metabolomic profiling analysis. Metabolomics 10, 1036–1041. 10.1007/s11306-014-0643-0 25254003 PMC4169990

[B137] More T. RoyChoudhury S. Gollapalli K. Patel S. K. Gowda H. Chaudhury K. (2015). Metabolomics and its integration with systems biology: PSI 2014 conference panel discussion report. J. Proteomics 127, 73–79. 10.1016/j.jprot.2015.04.024 25943869

[B138] Morris V. Rao X. Pickering C. Foo W. C. Rashid A. Eterovic K. (2017). Comprehensive Genomic Profiling of Metastatic Squamous Cell Carcinoma of the Anal Canal. Mol. Cancer Res. 15, 1542–1550. 10.1158/1541-7786.MCR-17-0060 28784613 PMC5991496

[B139] Murata T. Yanagisawa T. Kurihara T. Kaneko M. Ota S. Enomoto A. (2019). Salivary metabolomics with alternative decision tree-based machine learning methods for breast cancer discrimination. Breast Cancer Res. Treat. 177, 591–601. 10.1007/s10549-019-05330-9 31286302

[B140] Nam H. Chung B. C. Kim Y. Lee K. Lee D. (2009). Combining tissue transcriptomics and urine metabolomics for breast cancer biomarker identification. Bioinformatics 25, 3151–3157. 10.1093/bioinformatics/btp558 19783829

[B141] Nik-Zainal S. Davies H. Staaf J. Ramakrishna M. Glodzik D. Zou X. (2016). Landscape of somatic mutations in 560 breast cancer whole-genome sequences. Nature 534, 47–54. 10.1038/nature17676 27135926 PMC4910866

[B142] Nishiumi S. Kobayashi T. Ikeda A. Yoshie T. Kibi M. Izumi Y. (2012). A novel serum metabolomics-based diagnostic approach for colorectal cancer. PLoS One 7. 10.1371/journal.pone.0040459 PMC3394708 22792336

[B143] Osborne J. D. Wyatt M. Westfall A. O. Willig J. Bethard S. Gordon G. (2016). Efficient identification of nationally mandated reportable cancer cases using natural language processing and machine learning. J. Am. Med. Inf. Assoc. 23, 1077–1084. 10.1093/jamia/ocw006 PMC5070519 27026618

[B144] Paine M. R. L. Liu J. Huang D. Ellis S. R. Trede D. Kobarg J. H. (2019). Three-Dimensional Mass Spectrometry Imaging Identifies Lipid Markers of Medulloblastoma Metastasis. Sci. Rep. 9, 2205. 10.1038/s41598-018-38257-0 30778099 PMC6379434

[B145] Palubeckaitė I. Crooks L. Smith D. P. Cole L. M. Bram H. Le Maitre C. (2020). Mass spectrometry imaging of endogenous metabolites in response to doxorubicin in a novel 3D osteosarcoma cell culture model. J. Mass Spectrom. 55, e4461. 10.1002/jms.4461 31654532

[B146] Paolillo C. Londin E. Fortina P. (2016). Next generation sequencing in cancer: opportunities and challenges for precision cancer medicine. Scand. J. Clin. Lab. Invest. Suppl. 245, S84–S91. 10.1080/00365513.2016.1210331 27542004

[B147] Park K. Ali A. Kim D. An Y. Kim M. Shin H. (2013). Robust predictive model for evaluating breast cancer survivability. Eng. Appl. Artif. Intell. 26, 2194–2205. 10.1016/j.engappai.2013.06.013

[B148] Patel S. K. Rajora N. Kumar S. Sahu A. Kochar S. K. Krishna C. M. (2019). Rapid Discrimination of Malaria- and Dengue-Infected Patients Sera Using Raman Spectroscopy. Anal. Chem. 91, 7054–7062. 10.1021/acs.analchem.8b05907 31033270

[B149] Peng L. Cantor D. II Huang C. Wang K. Baker M. S. Nice E. C. (2018). Tissue and plasma proteomics for early stage cancer detection. Mol. Omi. 14, 405–423. 10.1039/c8mo00126j 30251724

[B150] Perakakis N. Yazdani A. Karniadakis G. E. Mantzoros C. (2018). Omics, big data and machine learning as tools to propel understanding of biological mechanisms and to discover novel diagnostics and therapeutics. Metabolism 87, A1–A9. 10.1016/j.metabol.2018.08.002 30098323 PMC6325641

[B151] Perng W. Rifas-Shiman S. L. McCulloch S. Chatzi L. Mantzoros C. Hivert M. F. (2017). Associations of cord blood metabolites with perinatal characteristics, newborn anthropometry, and cord blood hormones in project viva. Metabolism 76, 11–22. 10.1016/j.metabol.2017.07.001 28987236 PMC5675164

[B152] Poirion O. B. Chaudhary K. Huang S. Garmire L. X. (2019). Multi-omics-based pan-cancer prognosis prediction using an ensemble of deep-learning and machine-learning models. medRxiv, 19010082. 10.1101/19010082 PMC8281595 34261540

[B153] Porta Siegel T. Hamm G. Bunch J. Cappell J. Fletcher J. S. Schwamborn K. (2018). Mass Spectrometry Imaging and Integration with Other Imaging Modalities for Greater Molecular Understanding of Biological Tissues. Mol. Imaging Biol. 20, 888–901. 10.1007/s11307-018-1267-y 30167993 PMC6244545

[B154] Rai V. Karthikaichamy A. Das D. Noronha S. Wangikar P. P. Srivastava S. (2016). Multi-omics Frontiers in Algal Research: Techniques and Progress to Explore Biofuels in the Postgenomics World. OMICS 20, 387–399. 10.1089/omi.2016.0065 27315140

[B155] Ramazzotti D. Lal A. Wang B. Batzoglou S. Sidow A. (2018). Multi-omic tumor data reveal diversity of molecular mechanisms that correlate with survival. Nat. Commun. 9, 4453. 10.1038/s41467-018-06921-8 30367051 PMC6203719

[B156] Ribli D. Horváth A. Unger Z. Pollner P. Csabai I. (2018). Detecting and classifying lesions in mammograms with Deep Learning. Sci. Rep. 8, 4165. 10.1038/s41598-018-22437-z 29545529 PMC5854668

[B157] Robinson D. Van Allen E. M. Wu Y. M. Schultz N. Lonigro R. J. Mosquera J. M. (2015). Integrative clinical genomics of advanced prostate cancer. Cell 161, 1215–1228. 10.1016/j.cell.2015.05.001 26000489 PMC4484602

[B158] Robinson D. R. Wu Y.-M. Lonigro R. J. Vats P. Cobain E. Everett J. (2017). Integrative clinical genomics of metastatic cancer. Nature 548, 297–303. 10.1038/nature23306 28783718 PMC5995337

[B159] Romo-Bucheli D. Janowczyk A. Gilmore H. Romero E. Madabhushi A. (2017). A deep learning based strategy for identifying and associating mitotic activity with gene expression derived risk categories in estrogen receptor positive breast cancers. Cytometry A 91, 566–573. 10.1002/cyto.a.23065 28192639 PMC6124660

[B160] Rubio-Perez C. Tamborero D. Schroeder M. P. Antolín A. A. Deu-Pons J. Perez-Llamas C. (2015). In Silico Prescription of Anticancer Drugs to Cohorts of 28 Tumor Types Reveals Targeting Opportunities. Cancer Cell 27, 382–396. 10.1016/j.ccell.2015.02.007 25759023

[B161] Sakellaropoulos T. Vougas K. Narang S. Koinis F. Kotsinas A. Polyzos A. (2019). A Deep Learning Framework for Predicting Response to Therapy in Cancer. Cell Rep. 29, 3367–3373.e4. 10.1016/j.celrep.2019.11.017 31825821

[B162] Saltz J. Gupta R. Hou L. Kurc T. Singh P. Nguyen V. (2018). Spatial Organization and Molecular Correlation of Tumor-Infiltrating Lymphocytes Using Deep Learning on Pathology Images. Cell Rep. 23, 181–193.e7. 10.1016/j.celrep.2018.03.086 29617659 PMC5943714

[B163] Schoof E. M. Rapin N. Savickas S. Gentil C. Lechman E. Haile J. S. (2019). A Quantitative Single-Cell Proteomics Approach to Characterize an Acute Myeloid Leukemia Hierarchy. bioRxiv, 745679. 10.1101/745679

[B164] Sevakula R. K. Singh V. Verma N. K. Kumar C. Cui Y. (2019). Transfer Learning for Molecular Cancer Classification Using Deep Neural Networks. IEEE/ACM Trans. Comput. Biol. Bioinforma. 16, 2089–2100. 10.1109/TCBB.2018.2822803 29993662

[B165] Sirinukunwattana K. Ahmed Raza S. E. Tsang Y.-W. Snead D. R. J. Cree I. A. Rajpoot N. M. (2016). Locality Sensitive Deep Learning for Detection and Classification of Nuclei in Routine Colon Cancer Histology Images. IEEE Trans. Med. Imaging 35, 1196–1206. 10.1109/TMI.2016.2525803 26863654

[B166] Sohni A. Bartoccetti M. Khoueiry R. Spans L. Vande Velde J. De Troyer L. (2015). Dynamic Switching of Active Promoter and Enhancer Domains Regulates Tet1 and Tet2 Expression during Cell State Transitions between Pluripotency and Differentiation. Mol. Cell. Biol. 35, 1026–1042. 10.1128/mcb.01172-14 25582196 PMC4333094

[B167] Stemke-Hale K. Gonzalez-Angulo A. M. Lluch A. Neve R. M. Kuo W.-L. Davies M. (2008). An integrative genomic and proteomic analysis of PIK3CA, PTEN, and AKT mutations in breast cancer. Cancer Res. 68, 6084–6091. 10.1158/0008-5472.CAN-07-6854 18676830 PMC2680495

[B168] Stoeckli M. Chaurand P. Hallahan D. E. Caprioli R. M. (2001). Imaging mass spectrometry: A new technology for the analysis of protein expression in mammalian tissues. Nat. Med. 7, 493–496. 10.1038/86573 11283679

[B169] Sun D. Wang M. Li A. (2018). A multimodal deep neural network for human breast cancer prognosis prediction by integrating multi-dimensional data. IEEE/ACM Trans. Comput. Biol. Bioinforma. 16, 841–850. 10.1109/TCBB.2018.2806438 29994639

[B170] Sun C. Li T. Song X. Huang L. Zang Q. Xu J. (2019a). Spatially resolved metabolomics to discover tumor-associated metabolic alterations. Proc. Natl. Acad. Sci. U. S. A. 116, 52–57. 10.1073/pnas.1808950116 30559182 PMC6320512

[B171] Sun N. Kunzke T. Sbiera S. Kircher S. Feuchtinge A. Aichler M. (2019b). Prognostic relevance of steroid sulfation in adrenocortical carcinoma revealed by molecular phenotyping using high-resolution mass spectrometry imaging. Clin. Chem. 65, 1276–1286. 10.1373/clinchem.2019.306043 31492715

[B172] Syrjala K. L. (2018). Opportunities for improving oncology care. Lancet Oncol. 19, 449. 10.1016/S1470-2045(18)30208-0 29611517 PMC5951621

[B173] Therapeutically Applicable Research to Generate Effective Treatments (TARGET Available at: https://ocg.cancer.gov/programs/target (Accessed March 24, 2020).

[B174] Thorsen S. F. Gromova I. Christensen I. J. Fredriksson S. Andersen C. L. Nielsen H. J. (2019). Gel-Based Proteomics of Clinical Samples Identifies Potential Serological Biomarkers for Early Detection of Colorectal Cancer. Int. J. Mol. Sci. 20. 10.3390/ijms20236082 PMC6929140 31810358

[B175] Torata N. Kubo M. Miura D. Ohuchida K. Mizuuchi Y. Fujimura Y. (2018). Visualizing energy charge in breast carcinoma tissues by MALDI mass-spectrometry imaging profiles of low-molecular-weight metabolites, in Anticancer Research (International Institute of Anticancer Research), 4267–4272. 10.21873/anticanres.12723 29970560

[B176] Tsai M.-H. Wu C.-C. Peng P.-H. Liang Y. Hsiao Y.-C. Chien K.-Y. (2012). Identification of secretory gelsolin as a plasma biomarker associated with distant organ metastasis of colorectal cancer. J. Mol. Med. (Berl). 90, 187–200. 10.1007/s00109-011-0817-4 21997591

[B177] Turajlic S. Sottoriva A. Graham T. Swanton C. (2019). Resolving genetic heterogeneity in cancer. Nat. Rev. Genet. 20, 404–416. 10.1038/s41576-019-0114-6 30918367

[B178] Turki T. Wei Z. Wang J. T. L. (2018). A transfer learning approach via procrustes analysis and mean shift for cancer drug sensitivity prediction. J. Bioinform. Comput. Biol. 16:1840014. 10.1142/S0219720018400140 29945499

[B179] Uchiyama Y. Hayasaka T. Masaki N. Watanabe Y. Masumoto K. Nagata T. (2014). Imaging mass spectrometry distinguished the cancer and stromal regions of oral squamous cell carcinoma by visualizing phosphatidylcholine (16:0/16:1) and phosphatidylcholine (18:1/20:4). Anal. Bioanal. Chem. 406 (5), 1307–1316. 10.1007/s00216-013-7062-3 23728729

[B180] Van Emon J. M. (2016). The Omics Revolution in Agricultural Research. J. Agric. Food Chem. 64, 36–44. 10.1021/acs.jafc.5b04515 26468989 PMC4714296

[B181] Vantaku V. Dong J. Ambati C. R. Perera D. Donepudi S. R. Amara C. S. (2019). Multi-omics Integration Analysis Robustly Predicts High-Grade Patient Survival and Identifies CPT1B Effect on Fatty Acid Metabolism in Bladder Cancer. Clin. Cancer Res. 25, 3689–3701. 10.1158/1078-0432.CCR-18-1515 30846479 PMC6571061

[B182] Varambally S. Dhanasekaran S. M. Zhou M. Barrette T. R. Kumar-Sinha C. Sanda M. G. (2002). The polycomb group protein EZH2 is involved in progression of prostate cancer. Nature 419, 624–629. 10.1038/nature01075 12374981

[B183] Vasudevan P. Murugesan T. (2018). Cancer Subtype Discovery Using Prognosis-Enhanced Neural Network Classifier in Multigenomic Data. Technol. Cancer Res. Treat. 17:1533033818790509. 10.1177/1533033818790509 30092720 PMC6088521

[B184] Veselkov K. A. Vingara L. K. Masson P. Robinette S. L. Want E. Li J. V. (2011). Optimized preprocessing of ultra-performance liquid chromatography/mass spectrometry urinary metabolic profiles for improved information recovery. Anal. Chem. 83, 5864–5872. 10.1021/ac201065j 21526840

[B185] Vidavsky N. Kunitake J. A. M. R. Diaz-Rubio M. E. Chiou A. E. Loh H. C. Zhang S. (2019). Mapping and Profiling Lipid Distribution in a 3D Model of Breast Cancer Progression. ACS Cent. Sci. 5, 768–780. 10.1021/acscentsci.8b00932 31139713 PMC6535773

[B186] Vougas K. Sakellaropoulos T. Kotsinas A. Foukas G. R. P. Ntargaras A. Koinis F. (2019). Machine learning and data mining frameworks for predicting drug response in cancer: An overview and a novel in silico screening process based on association rule mining. Pharmacol. Ther. 203, 107395. 10.1016/j.pharmthera.2019.107395 31374225

[B187] Wang M. Carver J. J. Phelan V. V. Sanchez L. M. Garg N. Peng Y. (2016). Sharing and community curation of mass spectrometry data with Global Natural Products Social Molecular Networking. Nat. Biotechnol. 34, 828–837. 10.1038/nbt.3597 27504778 PMC5321674

[B188] Wang S. Chen X. Luan H. Gao D. Lin S. Cai Z. (2016). Matrix-assisted laser desorption/ionization mass spectrometry imaging of cell cultures for the lipidomic analysis of potential lipid markers in human breast cancer invasion. Rapid Commun. Mass Spectrom. 30, 533–542. 10.1002/rcm.7466 26777684

[B189] Wang Z. Jensen M. A. Zenklusen J. C. (2016). “A practical guide to The Cancer Genome Atlas (TCGA),” in Methods in Molecular Biology (Humana Press Inc.), 111–141. 10.1007/978-1-4939-3578-9_6 27008012

[B190] Wang X. Han J. Hardie D. B. Yang J. Pan J. Borchers C. H. (2017). Metabolomic profiling of prostate cancer by matrix assisted laser desorption/ionization-Fourier transform ion cyclotron resonance mass spectrometry imaging using Matrix Coating Assisted by an Electric Field (MCAEF). Biochim. Biophys. Acta - Proteins Proteomics 1865, 755–767. 10.1016/j.bbapap.2016.12.012 28017863

[B191] Wang Y. Wang D. Ye X. Wang Y. Yin Y. Jin Y. (2019). A tree ensemble-based two-stage model for advanced-stage colorectal cancer survival prediction. Inf. Sci. (NY). 474, 106–124. 10.1016/j.ins.2018.09.046

[B192] Wheeler D. A. Srinivasan M. Egholm M. Shen Y. Chen L. McGuire A. (2008). The complete genome of an individual by massively parallel DNA sequencing. Nature 452, 872–876. 10.1038/nature06884 18421352

[B193] Wilks C. Cline M. S. Weiler E. Diehkans M. Craft B. Martin C. (2014). The Cancer Genomics Hub (CGHub): overcoming cancer through the power of torrential data. Database (Oxford) 2014. 10.1093/database/bau093 PMC4178372 25267794

[B194] Wu T. Dai Y. (2017). Tumor microenvironment and therapeutic response. Cancer Lett. 387, 61–68. 10.1016/j.canlet.2016.01.043 26845449

[B195] Wu C. Zhou F. Ren J. Li X. Jiang Y. Ma S. (2019). A selective review of multi-level omics data integration using variable selection. High-Throughput 8. 10.3390/ht8010004 PMC6473252 30669303

[B196] Wu P. Heins Z. J. Muller J. T. Katsnelson L. de Bruijn I. Abeshouse A. A. (2019). Integration and Analysis of CPTAC Proteomics Data in the Context of Cancer Genomics in the cBioPortal. Mol. Cell. Proteomics 18, 1893–1898. 10.1074/mcp.TIR119.001673 31308250 PMC6731080

[B197] Xu J. Xiang L. Liu Q. Gilmore H. Wu J. Tang J. (2016). Stacked sparse autoencoder (SSAE) for nuclei detection on breast cancer histopathology images. IEEE Trans. Med. Imaging 35, 119–130. 10.1109/TMI.2015.2458702 26208307 PMC4729702

[B198] Xu C. Zhou D. Luo Y. Guo S. Wang T. Liu J. (2017). Tissue and serum lipidome shows altered lipid composition with diagnostic potential in mycosis fungoides. Oncotarget 8, 48041–48050. 10.18632/oncotarget.18228 28624795 PMC5564624

[B199] Xu J. Wu P. Chen Y. Meng Q. Dawood H. Dawood H. (2019). A hierarchical integration deep flexible neural forest framework for cancer subtype classification by integrating multi-omics data. BMC Bioinf. 20, 527. 10.1186/s12859-019-3116-7 PMC6819613 31660856

[B200] Yamazaki Y. (2015). Metabolome Analysis of Human Serum: Implications for Early Detection of Colorectal Cancer. Rinsho Byori. 63, 328–335. 26524856

[B201] Yang L. Rong W. Xiao T. Zhang Y. Xu B. Liu Y. (2013). Secretory/releasing proteome-based identification of plasma biomarkers in HBV-associated hepatocellular carcinoma. Sci. China Life Sci. 56, 638–646. 10.1007/s11427-013-4497-x 23749381

[B202] Yoon J. Jordon J. Van Der Schaar M. (2018). Ganite: Estimation of individualized treatment effects using generative adversarial nets. in 6th International Conference on Learning Representations, ICLR 2018 - Conference Track Proceedings.

[B203] Yu K.-H. Zhang C. Berry G. J. Altman R. B. Ré C. Rubin D. L. (2016). Predicting non-small cell lung cancer prognosis by fully automated microscopic pathology image features. Nat. Commun. 7, 12474. 10.1038/ncomms12474 27527408 PMC4990706

[B204] Yu K.-H. Berry G. J. Rubin D. L. Ré C. Altman R. B. Snyder M. (2017). Association of Omics Features with Histopathology Patterns in Lung Adenocarcinoma. Cell Syst. 5, 620–627.e3. 10.1016/j.cels.2017.10.014 29153840 PMC5746468

[B205] Yu P. Wilhelm K. Dubrac A. Tung J. K. Alves T. C. Fang J. S. (2017). FGF-dependent metabolic control of vascular development. Nature 545, 224–241. 10.1038/nature22322 28467822 PMC5427179

[B206] Yuan Y. Failmezger H. Rueda O. M. Raza Ali H. Gräf S. Chin S. F. (2012). Quantitative image analysis of cellular heterogeneity in breast tumors complements genomic profiling. Sci. Transl. Med. 4, 157ra143–157ra143. 10.1126/scitranslmed.3004330 23100629

[B207] Zeng I. S. L. Lumley T. (2018). Review of statistical learning methods in integrated omics studies (An integrated information science). Bioinform. Biol. Insights 12. 10.1177/1177932218759292 PMC5824897 29497285

[B208] Zhang W. Li F. Nie L. (2010). Integrating multiple “omics” analysis for microbial biology: Application and methodologies. Microbiology 156, 287–301. 10.1099/mic.0.034793-0 19910409

[B209] Zhang L. Jin H. Guo X. Yang Z. Zhao L. Tang S. (2012). Distinguishing pancreatic cancer from chronic pancreatitis and healthy individuals by 1H nuclear magnetic resonance-based metabonomic profiles. Clin. Biochem. 45, 1064–1069. 10.1016/j.clinbiochem.2012.05.012 22613268

[B210] Zhang T. Wu X. Ke C. Yin M. Li Z. Fan L. (2013). Identification of potential biomarkers for ovarian cancer by urinary metabolomic profiling. J. Proteome Res. 12, 505–512. 10.1021/pr3009572 23163809

[B211] Zhang S. Xu Y. Hui X. Yang F. Hu Y. Shao J. (2017). Improvement in prediction of prostate cancer prognosis with somatic mutational signatures. J. Cancer 8, 3261–3267. 10.7150/jca.21261 29158798 PMC5665042

[B212] Zhang J. Bajari R. Andric D. Gerthoffert F. Lepsa A. Nahal-Bose H. (2019). The International Cancer Genome Consortium Data Portal. Nat. Biotechnol. 37, 367–369. 10.1038/s41587-019-0055-9 30877282

[B213] Zhang X. Zhang J. Sun K. Yang X. Dai C. Guo Y. (2019). “Integrated Multi-omics Analysis Using Variational Autoencoders: Application to Pan-cancer Classification,” in Proceedings - 2019 IEEE International Conference on Bioinformatics and Biomedicine, BIBM 2019, 765–769. 10.1109/BIBM47256.2019.8983228

[B214] Zhao M. Tang Y. Kim H. Hasegawa K. (2018). Machine Learning With K-Means Dimensional Reduction for Predicting Survival Outcomes in Patients With Breast Cancer. Cancer Inform. 17:1176935118810215. 10.1177/1176935118810215 30455569 PMC6238199

[B215] Zhu L. Luo W. Su M. Wei H. Wei J. Zhang X. (2013). Comparison between artificial neural network and Cox regression model in predicting the survival rate of gastric cancer patients. Biomed. Rep. 1, 757–760. 10.3892/br.2013.140 24649024 PMC3917700

[B216] Zhuang J. Tang X. Du Z. Yang M. Zhou Y. (2016). Prediction of biomarkers of therapeutic effects of patients with lung adenocarcinoma treated with gefitinib based on progression-free-survival by metabolomic fingerprinting. Talanta 160, 636–644. 10.1016/j.talanta.2016.08.007 27591660

